# HDAC6 involves in regulating the lncRNA-microRNA-mRNA network to promote the proliferation of glioblastoma cells

**DOI:** 10.1186/s13046-022-02257-w

**Published:** 2022-02-02

**Authors:** An-Chih Wu, Wen-Bin Yang, Kwang-Yu Chang, Jung-Shun Lee, Jing-Ping Liou, Ruei-Yuan Su, Siao Muk Cheng, Daw-Yang Hwang, Ushio Kikkawa, Tsung-I Hsu, Chih-Yang Wang, Wen-Chang Chang, Pin-Yuan Chen, Jian-Ying Chuang

**Affiliations:** 1 Graduate Institute of Medical Sciences, College of Medicine, Taipei Medical University, Taipei, Taiwan; 2 The Ph.D. Program for Neural Regenerative Medicine, Taipei Medical University, 250 Wuxing Street, Taipei, 11031 Taiwan; 3 TMU Research Center of Neuroscience, Taipei Medical University, Taipei, Taiwan; 4 National Institute of Cancer Research, National Health Research Institutes, Tainan, Taiwan; 5 Department of Neurosurgery, National Cheng Kung University Hospital, Tainan, Taiwan; 6 School of Pharmacy, Taipei Medical University, Taipei, Taiwan; 7 TMU Research Center of Cancer Translational Medicine, Taipei Medical University, Taipei, Taiwan; 8 Cell Physiology and Molecular Image Research Center, Wan Fang Hospital, Taipei Medical University, Taipei, Taiwan; 9 The Ph.D. Program for Cancer Molecular Biology and Drug Discovery, Taipei Medical University, Taipei, Taiwan; 10 Department of Neurosurgery, Keelung Chang Gung Memorial Hospital, Chang Gung University, 222 Mai-jin Road, Keelung, 20401 Taiwan; 11 Department of Biomedical Science and Environmental Biology, Kaohsiung Medical University, Kaohsiung, Taiwan

**Keywords:** Glioblastoma, Histone deacetylase 6, lncRNA *LINC00461*, RNA-binding proteins

## Abstract

**Background:**

Glioblastoma (GBM) is the most aggressive and lethal brain tumor. Although the histone deacetylase (HDAC)/transcription factor axis promotes growth in GBM, whether HDACs including HDAC6 are involved in modulating long non-coding RNAs (lncRNAs) to affect GBM malignancy remains obscure.

**Methods:**

Integrative analysis of microarray and RNA-seq was performed to identify lncRNAs governed by HDAC6. Half-life measurement and RNA-protein pull-down assay combined with isobaric tags for relative and absolute quantitation (iTRAQ)-based proteomic analysis were conducted to identify RNA modulators. The effect of *LINC00461* on GBM malignancy was evaluated using animal models and cell proliferation-related assays. Functional analysis of the *LINC00461* downstream networks was performed comprehensively using ingenuity pathway analysis and public databases.

**Results:**

We identified a lncRNA, *LINC00461*, which was substantially increased in stem-like/treatment-resistant GBM cells. *LINC00461* was inversely correlated with the survival of mice-bearing GBM and it was stabilized by the interaction between HDAC6 and RNA-binding proteins (RBPs) such as carbon catabolite repression—negative on TATA-less (CCR4-NOT) core exoribonuclease subunit 6 and fused in sarcoma. Targeting *LINC00461* using azaindolylsulfonamide, an HDAC6 inhibitor, decreased cell-division-related proteins via the lncRNA-microRNA (miRNA)-mRNA networks and caused cell-cycle arrest, thereby suppressing proliferation in parental and drug-resistant GBM cells and prolonging the survival of mice-bearing GBM.

**Conclusions:**

This study sheds light on the role of *LINC00461* in GBM malignancy and provides a novel therapeutic strategy for targeting the HDAC6/RBP/*LINC00461* axis and its downstream effectors in patients with GBM.

**Supplementary Information:**

The online version contains supplementary material available at 10.1186/s13046-022-02257-w.

## Background

Glioblastoma (GBM) has a poor prognosis and remains incurable, despite aggressive treatment options including surgery, radiation therapy, and first-line chemotherapy with the drug temozolomide (TMZ). Less than 5% of the patients with GBM survive for 5 years following diagnosis. Approximately 90% of patients suffer from disease relapse within 2 years of treatment, regardless of their initial response to prior therapy [1]. Previous studies have revealed that GBM frequently harbors different gene expression patterns, causing patients to exhibit diverse clinical characteristics, survival times, and responses to treatment [2, 3]. Therefore, understanding the molecular mechanisms of GBM and developing novel strategies for treating the disease are urgent.

Although GBM is characterized by chromosome 10q loss, aberrant expression of proteins, such as epidermal growth factor receptor amplification and *p16*^*INK4a*^ deletion, and gene mutations, such as *TP53*, *IDH1*, and *PTEN*, the molecular basis of the onset and progression of GBM malignancy is not fully understood [4, 5]. Because protein-coding genes account for < 2% of the whole genome, examining the impact of non-coding genes on the regulation of the glioma phenotype appears essential [6]. Long non-coding RNAs (lncRNAs) are defined as transcripts exceeding 200 base pairs in length; these molecules have a wide variety of biological functions [7]. Accumulating evidence has revealed that many dysregulated lncRNAs contribute to chemoresistance and poor prognostic features of GBM [8]. As a result, identification of lncRNA as a potential target for treating patients with GBM is urgently needed.

The structure of lncRNA is comparable to that of mRNA, with a 7mGpppG 5′ cap and 3′-polyadenosine tail. Similar to mRNA decay, lncRNA stabilization and degradation are regulated by microRNAs, decay-promoting RNA-binding proteins (RBPs), decapping enzymes, and deadenylases [9]. Our previous study has demonstrated that azaindolylsulfonamide (MPT0B291), a histone deacetylase (HDAC) inhibitor selective for HDAC6 (IC_50_ ≈ 0.0052 μM) [10, 11], induces cellular senescence in stem-like GBM cells and prolongs the survival of mice-bearing temozolomide (TMZ)-resistant xenografts through downregulation of Sp1 and its targeted genes associated with drug resistance [10]. Although HDAC6 is known to maintain the acetylation balance of histones and nonhistone substrates, such as α-tubulin, cortactin, HSP90, and a few transcriptional factors [12], whether it participates in the regulation of lncRNAs that affect tumorigenesis remains obscure.

In the current study, we used integrative microarray and RNA-seq analysis to investigate whether HDAC6 modulates lncRNAs. A highly conserved lncRNA, *LINC00461*, which functions as an essential regulator in glioma formation [13] and regulates the expressions of genes such as DNA topoisomerase II Alpha (*TOP2A*) [13], B-cell lymphoma 2 (*BCL2*) [14], and integrin β3 (*ITGB*3) [15], was identified to be remarkably downregulated by HDAC6 depletion. Our data highlight *LINC00461*-associated regulatory networks in GBM malignancies, including treatment resistance and cancer stemness, and provide a novel insight into targeting the HDAC6/RBP/*LINC00461* axis as a therapeutic approach for patients with GBM.

## Materials and methods

### Materials

MPT0B291 [11], synthesized by Prof. J. P Liou at Taipei Medical University, was dissolved in dimethyl sulfoxide (DMSO; Sigma-Aldrich Corp., St. Louis, MO, USA). Temozolomide (TMZ) and trichostatin A (TSA) were purchased from MedChemExpress (Monmouth Junction, NJ, USA) and Santa Cruz Biotechnology, Inc. (Dallas, TX, USA), respectively.

### Cell culture

Human GBM cell lines, including U87MG (ATCC HTB-14; American Type Culture Collection, Manassas, VA, USA) and A172 (ATCC CRL-1620), and patient-derived primary GBM (Pt#3 and Pt#5) along with their TMZ-resistant and stem-like cells, were cultured in respective media as described previously [16]. Procedures utilized for establishing TMZ-resistant GBM cells were mentioned previously [16]. To maintain TMZ-resistant cells, 50 μM TMZ was added into the culture medium, and their resistance characteristics were confirmed using colony-formation assay. Informed consent obtained from the patients followed the protocols (Nos. 201,006,011 and 201,402,018) approved by the Joint Institutional Review Board (JIRB) of Taipei Medical University (Taipei, Taiwan).

### Transient transfection

FLAG-tagged CNOT6 (Sino Biological, Inc., Beijing, China) or the empty vector with FLAG tag was transfected into U87MG cells using PolyJet (SignaGen Laboratories, Frederick, MD, USA).

### Stable cell line construction

The expression vector pcDNA3.1(−) carrying *LINC00461* inserts (MDBio, Taipei, Taiwan) was transformed into *Escherichia coli* directly, and the DNA extracted from ampicillin (Sigma-Aldrich Corp.)-resistant bacteria was amplified using PCR. Pt#3 cells transiently transfected with pcDNA3.1-*LINC00461* using PolyJet (SignaGen Laboratories). Two days after transfection, cells stably expressing pcDNA3.1-*LINC00461* were selected by treating with 0.5 mg/mL geneticin (G418; Thermo Fisher Scientific, Waltham, MA, USA).

### RNA interference

Parental and TMZ-resistant GBM cells (U87MG, A172, and Pt#3) were transfected with 5, 10, or 20 nM specific gene siRNA or control non-targeting siRNA (ON-TARGETplus siRNA-SMARTpool/non-targeting pool, Dharmacon, Inc., Lafayette, CO, USA) using lipofectamine 3000 (Thermo Fisher Scientific). Seventy-two hours after the transfection of siRNA, the total RNA was extracted from GBM cells using TRIzol reagent (Thermo Fisher Scientific). The effects of depletion were validated by RT-qPCR. Targeted sequences for each gene are listed in Table S1.

### Microarray analysis

Stem-like and TMZ-resistant U87MG cells were treated with 10 and 6 μM MPT0B291, respectively, for 1 day. U87MG cells were treated with si-Sp1 for 3 days. Gene expression analysis following total RNA extracted from these cells using TRIzol reagent (Thermo Fisher Scientific) was performed by Welgene Biotech (Taipei, Taiwan) using a SurePrint G3 Human Gene Expression 8x60K Microarray (Agilent Technologies, Inc., Santa Clara, CA, USA). The number of non-coding genes with the signal-to-noise-ratio (SNR) threshold of > 1.0 was identified.

### RNA-seq

RNA-seq was performed by Welgene Biotech. In brief, after HDAC6 or *LINC00461* depletion, respectively, in TMZ-resistant U87MG and parental U87MG cells, the total RNA was extracted using Trizol Reagent (Thermo Fisher Scientific). Purified RNA was quantified at OD260 nm using an ND-1000 spectrophotometer (NanoDrop Technologies, Inc. Wilmington, DE, USA) and qualified using a Bioanalyzer 2100 (Agilent Technologies, Inc.) with RNA 6000 LabChip kit (Agilent Technologies, Inc.). The RNA-Seq libraries were constructed using Agilent’s SureSelect Strand-Specific RNA Library Preparation Kit, followed by AMPure XP Beads (Beckman Coulter, Brea, CA, USA) size selection, and the sequence was directly determined using Illumina’s sequencing-by-synthesis technology.

### Quantitative reverse transcription PCR (RT-qPCR)

Total RNA was extracted from cells using TRIzol reagent (Thermo Fisher Scientific), followed by reverse transcription using SuperScript IV First-Strand Synthesis System (Thermo Fisher Scientific) and TaqMan™ MicroRNA Reverse Transcription Kit (Thermo Fisher Scientific), respectively, for lncRNA or mRNA and miRNA. qPCR analysis was performed using iTaq Universal SYBR Green Supermix (Bio-Rad Laboratories, Inc., Hercules, CA, USA) and TaqMan™ Small RNA Assays, respectively, for lncRNA or mRNA and miRNA on StepOnePlus Real-Time PCR System (Applied Biosystems Inc., Waltham, MA, USA). Relative expression levels of target genes were determined using the delta–delta Ct (2^–∆∆Ct^) method and normalized against the glyceraldehyde 3-phosphate dehydrogenase (GAPDH) mRNA and U6 snRNA levels, respectively, for lncRNA or mRNA and miRNA. Oligonucleotides used for TaqMan qPCR assays were purchased from Suu-Flower Co., Ltd. (Taichung City, Taiwan). The primer or probe sequences for each gene are listed in Table S2.

### Animal models

For the subcutaneous tumor model, 1 × 10^6^ GBM cells (U87MG and Pt#3) were implanted into both dorsal flanks of female nonobese diabetic/severe combined immunodeficiency (NOD/SCID) mice (8 weeks old, BioLASCO Co., Ltd., Taipei, Taiwan). Intraperitoneal administration with MPT0B291 (10 mg/kg) or vehicle (DMSO) every other day was initiated when the tumor volume reached a size of 10–20 mm^3^ after tumor implantation. Tumor volumes were measured using a caliper three times a week and calculated following the modified ellipsoidal formula: tumor volume = 1/2 (length × width^2^) [17]. Tumor weights were recorded at the end of the experiments. For the orthotopic tumor model, 5 × 10^5^ Pt#3 cells (with or without stable *LINC00461* expression) were injected into the right frontal brain area of male Bagg albino (BALB)/c nude mice (9 weeks old, BioLASCO Co., Ltd.) according to detailed procedures reported previously [18]. Intraperitoneal administration with MPT0B291 (10 mg/kg) or vehicle (DMSO) every other day was initiated 5 days after tumor inoculation. Mice weights were recorded three times a week. All protocols for animal experiments were approved by the Institutional Animal Care and Use Committee of the National Health Research Institute (IACUC, NHRI, Tainan, Taiwan). Animal experiments were conducted under the registration number (NHRI-IACUC-106010).

### RNA-in situ hybridization (ISH) and RNA-fluorescence in situ hybridization (FISH)

Digoxigenin (DIG)-labeled probes (Custom LNATM Detection Probes; Table S3) targeting *LINC0046*1 or scramble were purchased from Qiagen (Hilden, Germany). In situ hybridization (ISH) was performed following the manufacturer’s instructions with slight modifications from IsHyb in situ hybridization kit (BioChain Institute Inc., Newark, CA, USA). In brief, 5-μm serial sections of MPT0B291-treated or control xenograft tumors were hybridized with DIG-labeled *LINC00461* or scramble probes (2–4 ng/μL) at 55 °C for 16 h. For ISH, chromogenic reaction used alkaline phosphatase (AP)-conjugated anti-DIG antibody (1:100) and 5-bromo-4-chloro-3-indolyl phosphate/nitro blue tetrazolium provided by manufacturer to detect the expression. For FISH, rhodamine tetramethylrhodamine-isothiocyanate-conjugated anti-DIG (111-025-003, 1:100, Jackson ImmunoResearch Laboratories, Inc., West Grove, PA, USA) was used to detect the expression instead. Cell nuclei were counterstained with nuclear fast red (Vector Laboratories, Inc., Burlingame, CA, USA) and 4′,6-diamidino-2-phenylindole (DAPI) (glycerol mounting medium - anti-fade with DAPI; ab188804, Abcam, Inc., Cambridge, UK), respectively, in ISH and FISH staining. Images of FISH staining were captured using ImageXpress Pico Automated Cell Imaging System (Molecular Devices, San Jose, CA, USA).

#### Immunohistochemistry

After antigen retrieval, immunohistochemistry was performed following the manufacturer’s instructions with slight modifications using VECTASTAIN® ABC AP Kits (Vector Laboratories, Inc.). In brief, 5-μm serial sections of MPT0B291-treated or control xenograft tumors were hybridized with an antibody against either Ki-67 (ab16667, 1:100, Abcam, Inc.) or cleaved caspase-3 (#9661, 1:100, Cell Signaling Technology, Inc., Danvers, MA, USA). Average 3,3′-diaminobenzidine staining intensities of each slide normalized by the nuclei number were calculated using the semi-quantitative method [19].

### mRNA stability measurement

Twenty-four hours after treating with MPT0B291 or vehicle control (DMSO), 10 μg/mL actinomycin D (ActD; Selleckchem, Houston, TX, USA) was added to inhibit transcription in TMZ-resistant Pt#3 and Pt#5 cells. RT-qPCR was used to detect the half-life of *LINC00461* at 0, 20, 40, 60, and 120 min after transcriptional inhibition.

### Immunoprecipitation assay

The cell lysate was prepared using radioimmunoprecipitation assay buffer containing protease inhibitor (Roche, Basel, Switzerland) and then immunoprecipitated with 2 μg anti-HDAC6 (#7612, Cell Signaling Technology, Inc.) or IgG (sc-2025/2027, Santa Cruz Biotechnology, Inc.) at 4 °C overnight, followed by incubation with protein A agarose (Merck Millipore, Bedford, MA, USA) at 4 °C for 1 h. For the immunoprecipitation of FLAG-tagged CNOT6, anti-FLAG M2 affinity gel (Sigma-Aldrich Corp.) was used following the manufacturer’s instructions. The immune complexes were detected by Western blot analyses with anti-CNOT6 (sc-81,231, 1:1000, Santa Cruz Biotechnology, Inc.), anti-HDAC6 (#7612, 1:1000, Cell Signaling Technology, Inc.), and anti-acetyl-lysine (GTX80693, 1:1000, GeneTex Inc., Irvine, CA, USA) antibodies.

### RNA-protein pull-down assay

Circular plasmid, pcDNA3.1-*LINC00461*, was converted to a linear template using EcoRV-HF digestion (New England Biolabs Inc., Ipswich, MA, USA) for in vitro *LINC00461* synthesis (HiScribe™ T7 Quick High Yield RNA Synthesis Kit; New England Biolabs). The pull-down of RNA-protein complexes was conducted using Pierce™ Magnetic RNA-Protein Pull-Down Kit (Thermo Fisher Scientific) based on the manufacturer’s instructions. Briefly, 50 pmol desthiobiotin-labeled (Pierce™ RNA 3′ End Biotinylation Kit, Thermo Fisher Scientific) androgen receptor (AR) 3′-untranslated regions (UTR) and *LINC00461* were captured by 50 μL streptavidin magnetic beads and then interacted with 200 μg lysate extracted from parental and TMZ-resistant Pt#3 cells. Elution of RNA-binding protein complexes was applied for western blotting and isobaric tags for relative and absolute quantitation (iTRAQ)-based proteomic analysis (Biotools Co. Ltd., New Taipei City, Taiwan). 3′-UTR of AR RNA, which contains UC-rich regions for human antigen R binding, is the control system for the pull-down assay.

### Cell proliferation assay

Parental and TMZ-resistant cells (U87MG and A172) at a density of 10^4^ cells per well were seeded in 24-well tissue culture plates overnight. After downregulation of *LINC00461* using siRNA, cell proliferation was determined by adding 0.5 mg/mL 3-(4,5-dimethylthiazol-2-yl)-2,5-diphenyltetrazolium bromide (MTT, BIONOVAS Biotechnology Co., Ltd., Toronto, CA) for 30 min at 37 °C. After the reaction, the formazan products were dissolved in 0.3 mL DMSO (Sigma-Aldrich Corp.), and the absorbance was spectrophotometrically measured at 550 nm every 24 h by an ELIZA reader (Bio-Rad Laboratories, Inc.).

### Cell-cycle analysis

The cell cycle was evaluated by flow cytometry. Briefly, parental and TMZ-resistant U87MG cells at a density of 7.5 × 10^4^ cells per well were seeded in 6-cm petri dishes. After downregulation of *LINC00461* using siRNA, cells were collected and fixed with 70% ethanol at 4 °C overnight. Fixed cells were incubated with 0.5 mL PBS containing 0.1% Triton X-100 (Pharmacia & Upjohn Company LLC, North Peapack, NJ, USA) on ice for 15 min and then stained with propidium iodide (Sigma-Aldrich Corp.)/RNase A (BIONOVAS Biotechnology Co.) solution (20 μg/mL propidium iodide and 0.3 mg/mL RNase A in PBS) for 30 min in darkness. Stained cells were measured by flow cytometry (Guava easyCyte, Merck Millipore). Cell-cycle distribution was analyzed by calculating percentages of cells at subG1, G0/G1, S, and G2/M phases.

### Western blot analysis

For western blot analysis, anti-Sp1 (#07-645, 1:1000, MilliporeSigma, Burlington, MA, USA), anti-HDAC1 (H3284, 1:1000, MilliporeSigma), anti-HDAC2 (#05-814, 1:1000, MilliporeSigma), anti-HDAC6 (#7612, 1:1000, Cell Signaling Technology, Inc.), anti-CNOT6 (sc-81,231, 1:1000, Santa Cruz Biotechnology, Inc.), anti-fused in sarcoma (FUS) (A5921, 1:1000, ABclonal Inc., Woburn, MA, USA), anti-Acetyl-lysine (GTX80693, 1:1000, GeneTex Inc.), anti-PABP (ab21060, 1:1000, Abcam, Inc.), anti-TOP2A (MAB4197, 1:1000, MilliporeSigma), anti-Ki67 (A11005, 1:1000, ABclonal Inc.), anti-MELK (A3530, 1:1000, ABclonal Inc.), anti-DLGAP5 (A2197, 1:1000, ABclonal Inc.), anti-CD168 (A11666, 1:1000, ABclonal Inc.), anti-MCM10 (A5427, 1:1000, ABclonal Inc.), anti-TMPO (A2534, 1:1000, ABclonal Inc.), anti-cyclin D1 (E1A6234, 1:1000, EnoGene Biotech Co., Ltd., New York, NY, USA), anti-CDK1 (sc-8395, 1:1000, Santa Cruz Biotechnology, Inc.), and anti-alpha Tubulin (66031-1-Ig, 1:10,000, Proteintech Group, Inc., Rosemont, IL, USA) were used as primary antibodies. HRP-linked anti-mouse IgG (sc-2004, 1:5000, Santa Cruz Biotechnology, Inc.) and HRP-linked anti-rabbit IgG (sc-2005, 1:5000, Santa Cruz Biotechnology, Inc.) were used as secondary antibodies. The signals were amplified using an enhanced chemiluminescence reagent (ECL, GE Healthcare, Chicago, IL, USA) and captured using the ChemiDoc Touch Imaging System (Bio-Rad Laboratories, Inc.). The intensity of each band was determined by Image Lab, and the relative target protein levels were normalized against the tubulin level.

### Single-cell RNA sequencing (scRNA-seq)

Fresh tumor tissues from four patients with GBM were prepared for scRNA-seq following the protocol established by 10x Genomics® Single Cell (Pleasanton, CA, USA). Data analysis was performed using Cell Ranger (10x Genomics®) and Seurat (29608179). Informed consent obtained from the patients followed a protocol (IRB No. EC1080202) approved by the Research Ethics Committee of the National Health Research Institute (NHRI, Miaoli, Taiwan).

### Analysis of databases

Next-generation sequencing (NGS) databases of human GBM transcriptome obtained from The Cancer Genome Atlas (TCGA) (https://portal.gdc.cancer.gov) were collected and processed as described previously [10]. The expression fold change and significance level (*t*-test) of 50 cell-division-related genes, which appeared in the intersection dataset of *LINC00461* depletion and MPT0B291 treatment, between normal and GBM NGS data were calculated. The correlation of gene expression status between TCGA-GBM and *LINC00461*-silenced datasets was computed using Pearson’s correlation coefficient (PCC). Prognostic implications of cell-division-related genes in high-grade glioma patients were analyzed using PROGgeneV2 (http://www.progtools.net/gene/) [20]. Three public databases, miRDB (http://mirdb.org/), miRWALK (http://mirwalk.umm.uni-heidelberg.de/), and starBase (http://starbase.sysu.edu.cn/index.php) were used to predict the lncRNA-miRNA-mRNA networks.

### Bioinformatic analyses

The heatmap, which displayed the log2 (fold change) values of selected lncRNAs from microarray datasets, was generated using online software Morpheus (https://software.broadinstitute.org/morpheus). Functional analysis of data from microarray and RNA sequencing was performed using ingenuity pathway analysis (IPA, Qiagen). The overlap between molecules in our datasets and a particular function was calculated using the right-tailed Fisher’s exact test. Prediction of activation or inhibition of pathways was examined using a Z-score. The heat map was generated using a MultiExperiment Viewer by the log2 (fold change) values of selected genes.

### Statistics

The statistical significance of the difference between the two groups was analyzed using the unpaired Student’s *t*-test with a two-tailed *p*-value. For more than two groups, one-way or two-way analysis of variance (ANOVA) was used to examine statistically significant differences depending on the number of variables. The survival curves of different groups were compared using the log-rank test. A *p*-value of < 0.05 was considered statistically significant. The statistical significance increased in the order: ^*^p < 0.05, ^**^p < 0.01, ^***^p < 0.001, and ^****^p < 0.0001.

## Results

### HDAC6 inhibition significantly decreases *LINC00461* expression

**Fig. 1:**
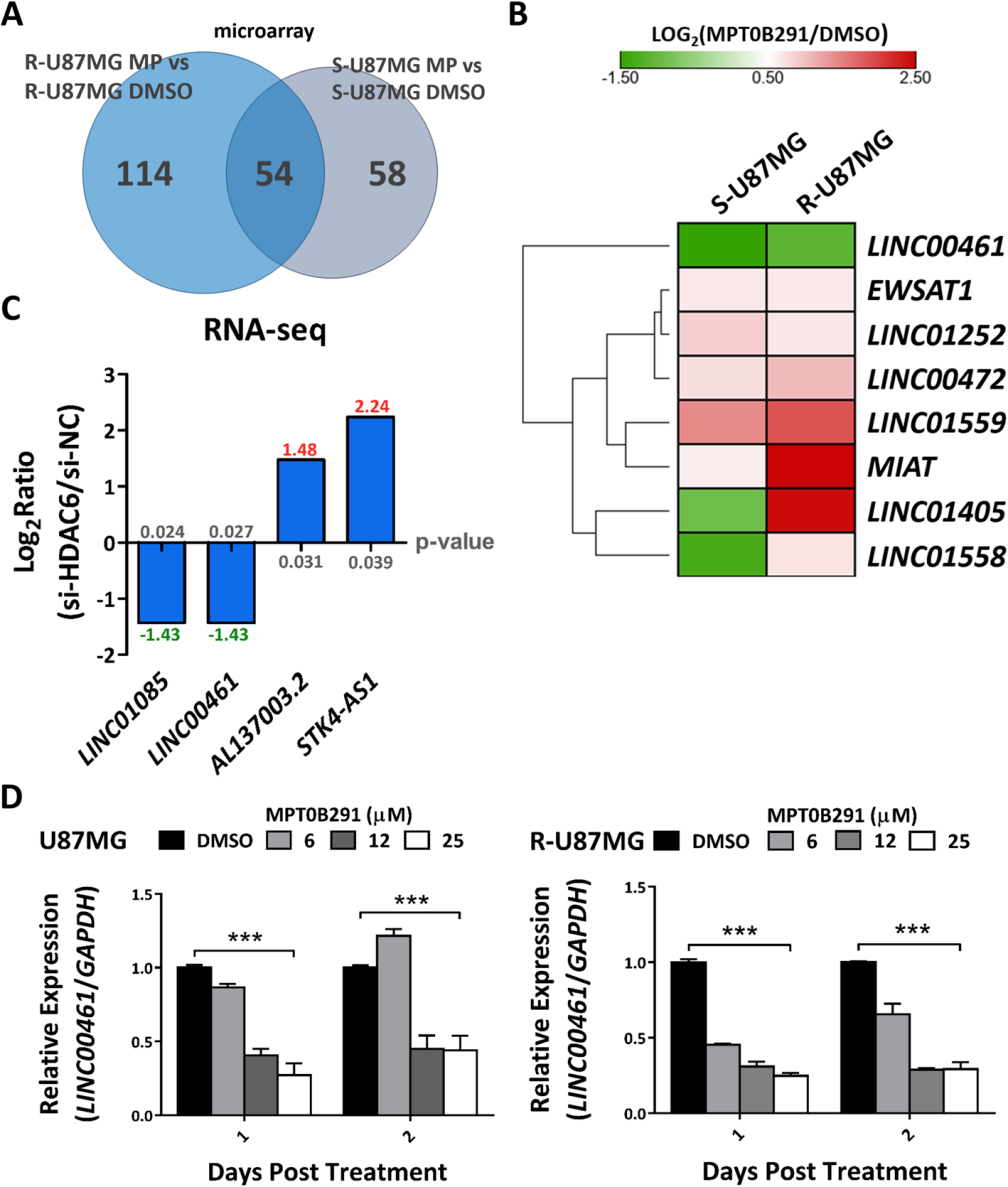
Significant reduction of *LINC00461* expression in response to MPT0B291 treatment in glioblastomas (GBMs). **A** Venn diagram depicts the number of differentially expressed lncRNAs selected by microarray analysis in stem-like (S-U87MG) and temozolomide (TMZ)-resistant (R-U87MG) GBM cells treated with MPT0B291 (MP, 10 μM in S-U87MG; 6 mM in R-U87MG) and DMSO, respectively, for 1 day. **B** Heatmap represents the significantly changed expression of long non-coding RNAs (lncRNAs; fold change ≥1.5) by MPT0B291 in the two microarray datasets in (**A**). The color scale indicates the relative fold change (log2) to control (DMSO) of each lncRNA, where red represents high expression and green represents low expression. Genes are hierarchically clustered based on their expression values. **C** RNA-seq revealed the discriminative expression of lncRNAs post HDAC6 knockdown. Y-axis represents relative expression level (log2) to control (si-NC) of each lncRNA shown in the x-axis. **D** Effect of MPT0B291 on *LINC00461* expression in parental and TMZ-resistant U87MG cells after 1-2 days of treatment. The results are shown as mean ± standard error of the mean (SEM) for triplicate samples in each group. One-way ANOVA

To determine whether HDAC6 affects lncRNA regulation, we analyzed the expression levels of 9445 lncRNAs using microarray. Microarray gene expression data were applied independently on mRNA samples isolated from GBM stem-like cells (serum-free/suspension-adapted tumorspheres) [21] and TMZ-resistant cells [18]. A comparison of MPT0B291-treated with DMSO-treated conditions showed that 54 lncRNA probe sets represent reliable microarray signals (signal-to-noise ratio ≥ 1, Fig. 1A). Among these, only two lncRNAs (*LINC00461* and *LINC01559*) were commonly and significantly altered in stem-like (S-U87MG) and TMZ-resistant (R-U87MG) GBM cells following MPT0B291 treatment (Fig. 1B, Fig. S1). *LINC00461* levels were decreased 0.27-fold and 0.49-fold in S- and R-U87MG cells, respectively, while *LINC01559* levels were upregulated 2.70-fold and 3.64-fold in S- and R-U87MG cells, respectively. To further confirm the effect of HDAC6 on lncRNA regulation, we depleted HDAC6 using siRNA and performed RNA-seq analysis. Four lncRNAs showing altered levels after HDAC6 knockdown were identified (Fig. 1C). Interestingly, a comparison of datasets obtained from MPT0B291 treatment and HDAC6 knockdown revealed that *LINC00461* was the only lncRNA with an obvious reduction after HDAC6 inhibition, suggesting that it is a downstream lncRNA target of HDAC6. Moreover, further confirmation using RT-qPCR analysis showed a significant reduction in *LINC00461* levels by MPT0B291 treatment in both parental and TMZ-resistant U87MG cells (Fig. 1D).

### Attenuation of *LINC00461* by MPT0B291 remarkably extends the survival time of mice with GBM

**Fig. 2:**
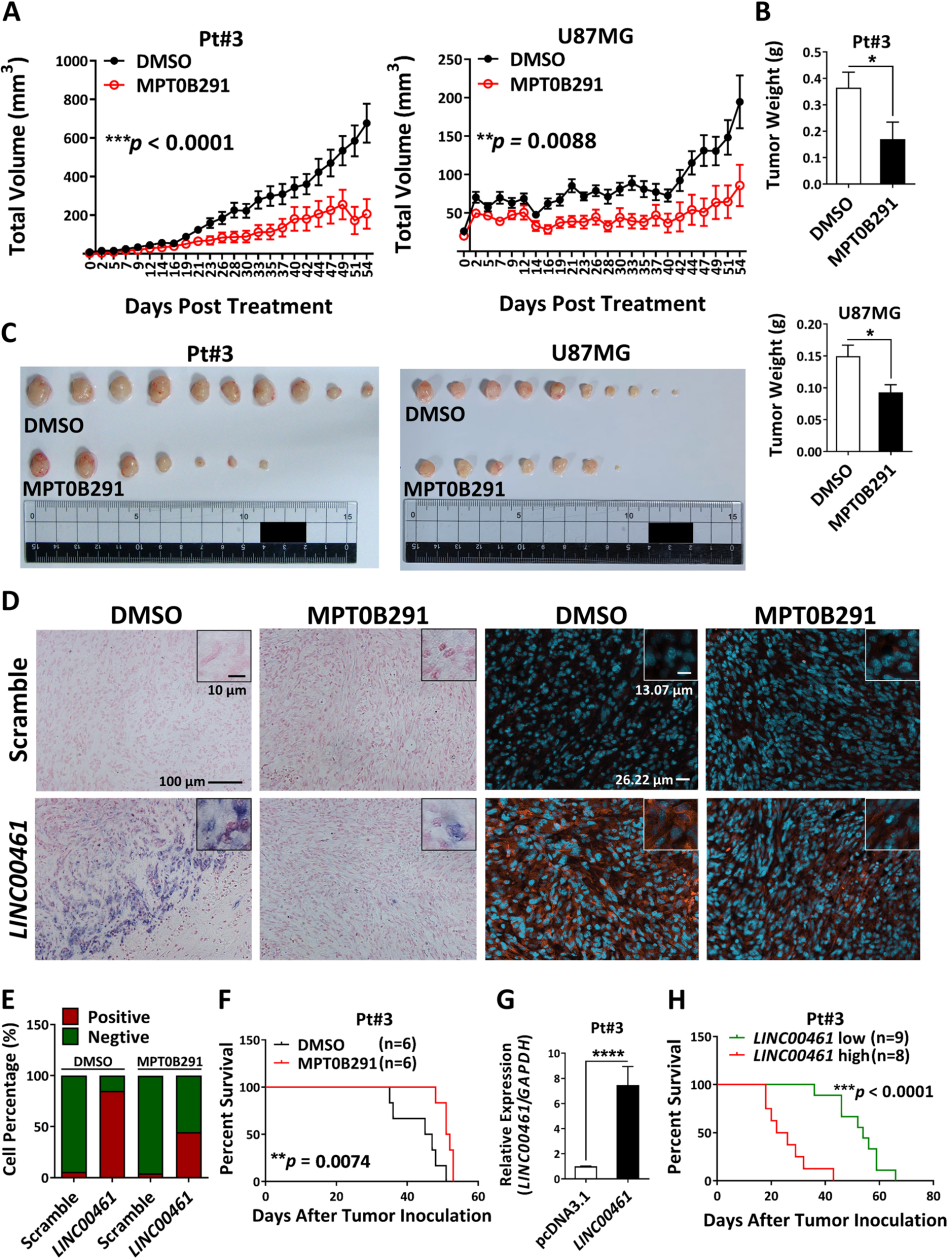
Suppression of *LINC00461* by MPT0B291 inhibits tumor growth and improves survival of mice-bearing intracranial GBM. **A** Growth curves show average tumor volumes from 1 × 10^6^ Pt#3 cells and 1 × 10^6^ U87MG cells injected into the dorsal flanks of NOD/SCID mice. MPT0B291 was administered after the tumor volume reached a size of 10–20 mm^3^ every other day (*n* ≥ 5 per group). Two-way ANOVA. **B** Average final tumor weights of control and MPT0B291-treated xenografts (n ≥ 5 per group). Unpaired Student’s *t*-test. **C** Images of control and MPT0B291-treated xenografts at the end of the experiment (n ≥ 5 per group). **D** ISH and FISH detected the expression of *LINC00461*. Serial sections of control and MPT0B291-treated xenografts were hybridized with either a *LINC00461* probe or a scramble probe in ISH and FISH, and cell nuclei were stained with nuclear fast red and DAPI, respectively, in ISH (left 4 panels) and FISH (right 4 panels) staining. Scale bars indicate 10 μm (inset of ISH), 100 μm (ISH), 13.07 μm (inset of FISH), and 26.22 μm (FISH). **E** Relative percentages of *LINC00461* expression in the cells of control and MPT0B291-treated xenografts were measured by fluorescent intensity. **F** The Kaplan–Meier plot represents the survival time of BALB/c nude mice-bearing orthotopically implanted 5 × 10^5^ Pt#3 cells. MPT0B291 (10 mg/kg) was administered 5 d after cell implantation every other day (*n* = 6 per group). Log-rank test. **G** The expression levels of *LINC00461* in Pt#3 cells stably expressing either *LINC00461 or* empty vector control (pcDNA3.1). Unpaired Student’s *t*-test. **H** The Kaplan–Meier plot represents the survival time of BALB/c nude mice-bearing orthotopically implanted 5 × 10^5^ Pt#3 cells stably expressing either *LINC00461 or* empty vector control (pcDNA3.1) (*n* = 8–9 per group). Log-rank test. The results are shown as mean ± SEM for triplicate samples in each group

Using U87MG xenografts and Pt#3 xenografts in mice, we observed a significant reduction in tumor growth after MPT0B291 treatment (Fig. 2A–C). Moreover, we analyzed the expression of *LINC00461* in MPT0B291-treated and control xenografts by RNA ISH and RNA FISH. A decrease of approximately 40% in *LINC00461* molecules was visualized within the cytoplasm of MPT0B291-treated xenograft tissues compared to that in control tumors (Fig. 2D–E), confirming the suppressive effect of MPT0B291 on *LINC00461* expression in vivo. Furthermore, MPT0B291 treatment extended the survival of mice-bearing intracranial GBM (Fig. 2F). To understand the mechanism by which MPT0B291 treatment prolonged survival through downregulating *LINC00461*, protein expressions of proliferation (Ki-67) and apoptosis (cleaved caspase-3) marker were measured in xenograft tissues. A significant reduction in Ki-67 expression was observed within the nucleus of MPT0B291-treated xenograft tissues compared to that in control tumors (Fig. S2A). In contrast, the difference in cleaved caspase-3 expression was not significant in the nucleus between MPT0B291-treated and control xenograft tissues (Fig. S2A), suggesting that the extension of survival in mice-bearing GBM is mainly derived from the anti-proliferative effect of MPT0B291. We then evaluated the role of *LINC00461* on survival outcomes using the same orthotopic mouse model. Pt#3 cells that stably express *LINC00461* were constructed (Fig. 2G) and injected into the right cerebral hemisphere of mice. The *LINC00461* high expression group’s median survival was 24 d, while that of the *LINC00461* low expression group was 54 d (Fig. 2H). Thus, *LINC00461* plays an important role in GBM growth, but HDAC6 inhibition induced by MPT0B291 blocks the oncogenic effect of *LINC00461*.

### HDAC6 and RNA-binding proteins are involved in managing *LINC00461* expression

**Fig. 3:**
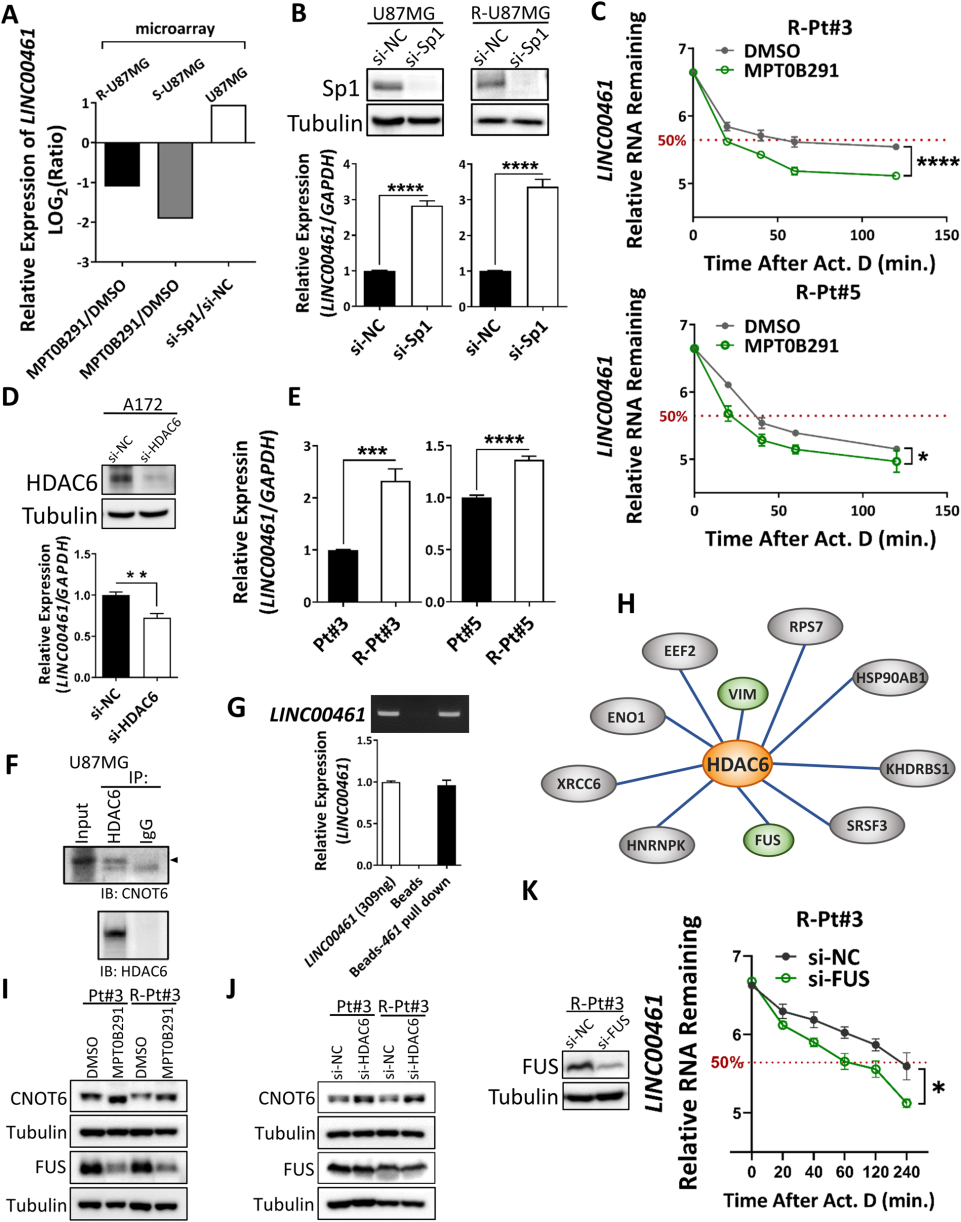
Interaction of HDAC6 and RBPs in regulating *LINC00461* stability. **A** Expression levels of *LINC00461* from transcriptome data, including two microarray data as shown in Fig. 1A and one microarray analysis using Sp1 knockdown cells. **B** Parental and TMZ-resistant U87MG cells were treated with either si-Sp1 to silence Sp1 expression or si-NC as a control. Sp1 protein levels were detected by western blotting. *LINC00461* expression was detected by qRT-PCR. Unpaired Student’s *t*-test. **C** The amount of *LINC00461* was measured in TMZ-resistant Pt#3 and Pt#5 cells in either the presence (green lines) or the absence (gray lines) of MPT0B291 following actinomycin D (ActD) treatment at 0, 20, 40, 60, and 120 min. Two-way ANOVA. The red dashed line indicates the time at which 50% of the lncRNA remained. **D** A172 cells were treated with either si-HDAC6 to silence HDAC6 expression or si-NC as a control. HDAC6 protein levels were detected by western blotting. *LINC00461* expression was detected by qRT-PCR. Unpaired Student’s *t*-test. **E** Expression levels of *LINC00461* in parental and TMZ-resistant GBM cells (Pt#3 and Pt#5). Unpaired Student’s *t*-test. **F** Ingenuity pathway analysis (IPA) in U87MG cells was performed using the antibody against HDAC6 or IgG as a negative control. The IP product was examined by western blotting with CONT6 and HDAC6 antibodies. **G** Desthiobiotin-labeled *LINC00461* was captured by streptavidin magnetic beads. PCR (upper panel) and qRT-PCR (lower panel) detected the amount of *LINC00461* binding to beads. **H** Proteins associated with *LINC00461* in TMZ-resistant Pt#3 cells were identified by iTRAQ-based proteomic analysis. Interaction between *LINC00461*-binding proteins and HDAC6 was further evaluated by IPA analysis. **I** Effect of MPT0B291 on the protein expression of RBPs (CNOT6 and FUS) at 48 h in parental and TMZ-resistant Pt#3 cells. **J** Effect of HDAC6 knockdown by siRNA on the protein expression of RBPs (CNOT6 and FUS) in parental and TMZ-resistant Pt#3 cells. The results are shown as mean ± SEM for triplicate samples in each group. **K** The amount of *LINC00461* was measured in TMZ-resistant Pt#3 cells treated with either si-FUS to silence FUS expression (green lines) or si-NC as a control (gray lines) following actinomycin D (ActD) treatment at 0, 20, 40, 60, 120, and 240 min. FUS protein levels were detected by western blotting. Two-way ANOVA. The red dashed line indicates the time at which 50% of lncRNA remained

MPT0B291 is known to block the activation of the HDAC6/Sp1 axis [10]. Whether MPT0B291 suppresses *LINC00461* expression by a Sp1-dependent mechanism was further investigated. The result showed that *LINC00461* expression was substantially decreased by MPT0B291 treatment while it increased with Sp1 depletion (Fig. 3A, bars 1–3). Consistently, RT-qPCR detection after Sp1 knockdown confirmed elevated *LINC00461* levels in parental and TMZ-resistant U87MG cells (Fig. 3B), suggesting that MPT0B291-mediated downregulation of *LINC00461* is modulated via an Sp1-independent pathway. Furthermore, to explore the mechanism underlying the modulation of *LINC00461* expression, we investigated the RNA stability of *LINC00461* using dactinomycin to block de novo RNA transcription in cells and found that MPT0B291 treatment caused 2.75-fold and 1.75-fold reduction in the half-life of *LINC00461* in TMZ-resistant Pt#3 and Pt#5 cells, respectively (Fig. 3C). Considering that MPT0B291 is a selective inhibitor of HDAC6 and based on HDAC6 knockdown reduction of *LINC00461* levels as verified by RNA-seq analysis (Fig. 1C) and RT-qPCR detection (Fig. 3D), HDAC6 was presumed to contribute to the regulation of *LINC00461* stability. Additionally, we found that *LINC00461* expression was higher in TMZ-resistant GBM cells. Increased expression of *LINC00461* may correspond with the malignant behavior of GBM cells.

HDACs are known to regulate post-transcriptional gene expression by altering deadenylase complex activation and affecting polyadenylate tail stability of RNA [22]. Thus, we examined HDAC6 interaction with the catalytic subunits of the deadenylase complex and identified that carbon catabolite repression—negative on TATA-less (CCR4-NOT) core exoribonuclease subunit 6 (CNOT6), a typical protein related to mRNA decay, is associated with HDAC6 in U87MG cells (Fig. 3F). Furthermore, treating cells with HDAC6 inhibitors, MP0B291 and trichostatin A, led to a decrease in the interaction of HDAC6 and CNOT6 in cells (Fig. S3A), suggesting that deacetylase activity is essential for HDAC6 to bind CNOT6, causing changes in the deadenylase activation. Apart from RNA-degrading enzymes that contribute to RNA degradation, RNA-protective proteins that contribute to the stability of *LINC00461* were also investigated. We purified *LINC00461* (Fig. 3G) and performed RNA-protein pull-down assay (Fig. S3B) combined with iTRAQ-based proteomic analysis, by which several *LINC00461*-binding proteins were identified (Fig. 3H). Further investigation of the proteomics data using IPA, two RNA-stabilizing proteins, FUS and vimentin, were discovered as HDAC6-targeted factors that functioned in regulating RNA stability. Interestingly, a previous study has indicated that FUS is associated with several RBPs in the vicinity of the mRNA 3′ end and it controls polyadenylate tail maintenance [23]. To investigate the role of HDAC6 on these RNA-regulating proteins, HDAC6 inhibition was induced by MPT0B291 treatment or HDAC6 knockdown and this caused a significant decrease in FUS expression in both parental and TMZ-resistant GBM cells and a significant increase in CNOT6 expression (Fig. 3I–J and Fig. S3C). Furthermore, the effect of FUS knockdown on the RNA stability of *LINC00461* showed a 3-fold reduction in the half-life of *LINC00461* in TMZ-resistant Pt#3 cells (Fig. 3K).

### *LINC00461* depletion suppresses GBM cell proliferation through cell-cycle arrest

**Fig. 4:**
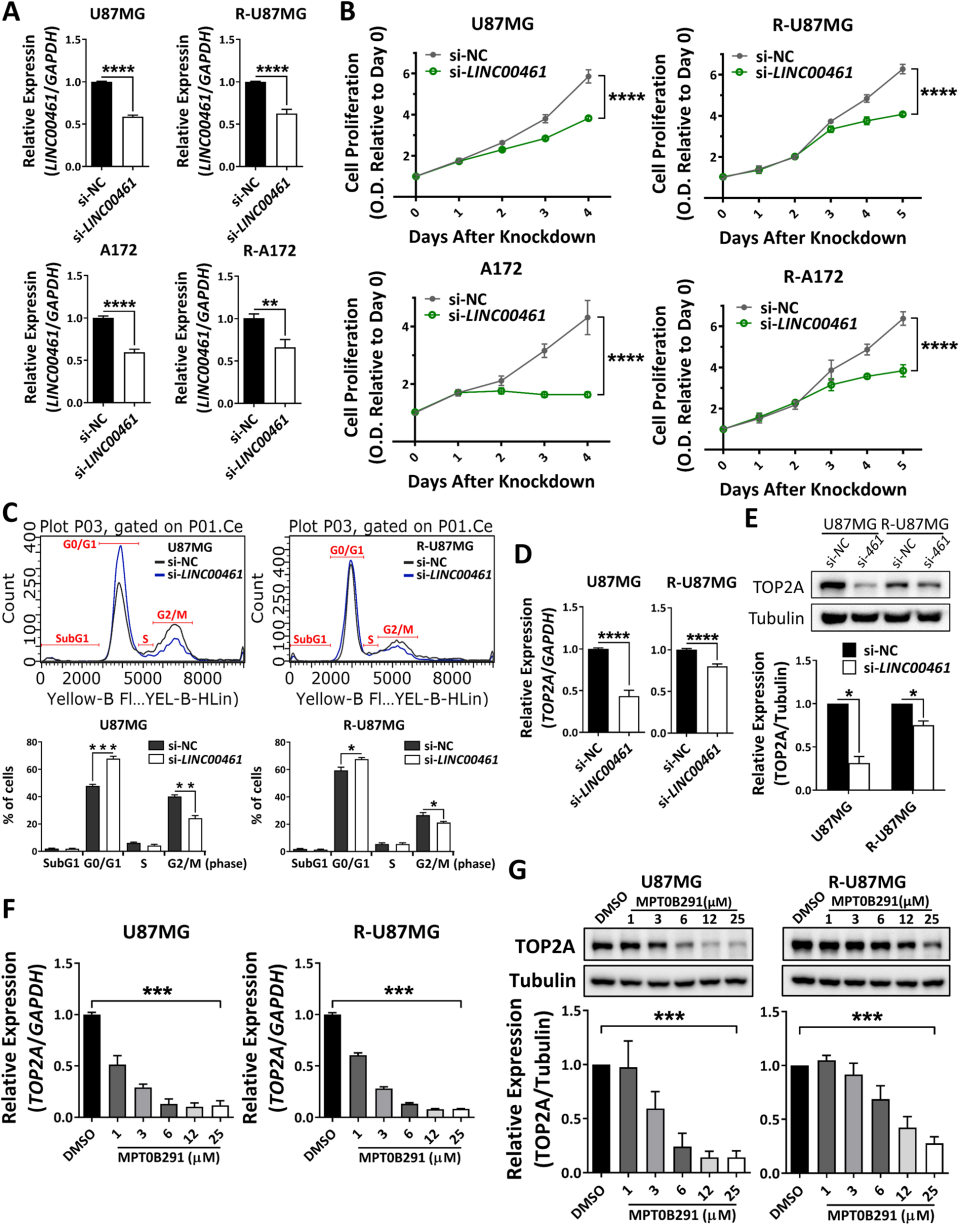
Inhibition of *LINC00461* downregulates TOP2A, resulting in the blocking of cell proliferation and cell-cycle progression in GBM cells. **A** Expression levels of *LINC00461* in parental and TMZ-resistant GBM cells (U87MG and A172) treated with either si-NC or si-*LINC00461*. Unpaired Student’s *t*-test. **B** Effect of *LINC00461* depletion on proliferation in parental and TMZ-resistant GBM cells (U87MG and A172). The x-axis indicates the time after siRNA transfection. OD_550_ values relative to si-NC on day 0 are shown in the y-axis. Two-way ANOVA. **C** Cells (U87MG and R-U87MG) with or without *LINC00461* knockdown were fixed and stained with propidium iodide, and then their DNA contents were measured by flow cytometry. X- and y-axes denote DNA content and cell number, respectively. The lower panel shows the quantitative measurements of each of cell-cycle phase. Unpaired Student’s *t*-test. **D** Expression of *TOP2A* mRNA in U87MG and R-U87MG cells that were treated with either si-NC or si-*LINC00461*. Unpaired Student’s *t*-test. **E** Cells (U87MG and R-U87MG) were treated with si-NC or si-*LINC00461*. After knockdown, TOP2A protein levels were detected by western blotting. Quantification of TOP2A expression from triplicate samples is shown below. Unpaired Student’s *t*-test. **F** and **G** Cells (U87MG and R-U87MG) were treated with different doses of MPT0B291 for 2 d, and the levels of TOP2A mRNA (**F**) and protein (**G**) in cells were then detected by qRT-PCR and western blotting, respectively. Quantification of mRNA and protein levels was obtained from triplicate samples. One-way ANOVA. The results are shown as mean ± SEM for triplicate samples in each group

We examined the effects of *LINC00461* on GBM cell proliferation. *LINC00461* knockdown in parental and TMZ-resistant GBM cells reduced *LINC00461* expression approximately by 40–50% (Fig. 4A). Interestingly, *LINC00461* depletion suppressed cell proliferation significantly in both parental and TMZ-resistant GBM cells (Fig. 4B). To further determine the anti-proliferative effect of *LINC00461* silencing on GBM cells, we analyzed cell-cycle distribution by flow cytometry. *LINC00461* knockdown induced a significant cell-cycle shift from G2/M to G1 phases in parental GBM cells and similarly induced a slight perturbation of cell-cycle progression into G2/M phase in TMZ-resistant cells (Fig. 4C). Recently, topoisomerase II alpha (TOP2A), a key enzyme in DNA replication to maintain cell proliferation, was found to be upregulated by *LINC00461* through sponging of miR-411-5p during gliomagenesis [13]. Thus, we examined TOP2A expression, including levels of RNA (Fig. 4D) and protein (Fig. 4E), in our experimental model. The results showed that *LINC00461* knockdown significantly attenuated TOP2A expression at the transcriptional level in both parental and TMZ-resistant U87MG cells. Additionally, MPT0B291 treatment showed a similar trend with reduced mRNA and protein expression of TOP2A in these GBM cells (Fig. 4F–G).

### *LINC00461* is involved in HDAC6-mediated cell division and survival regulation

**Fig. 5:**
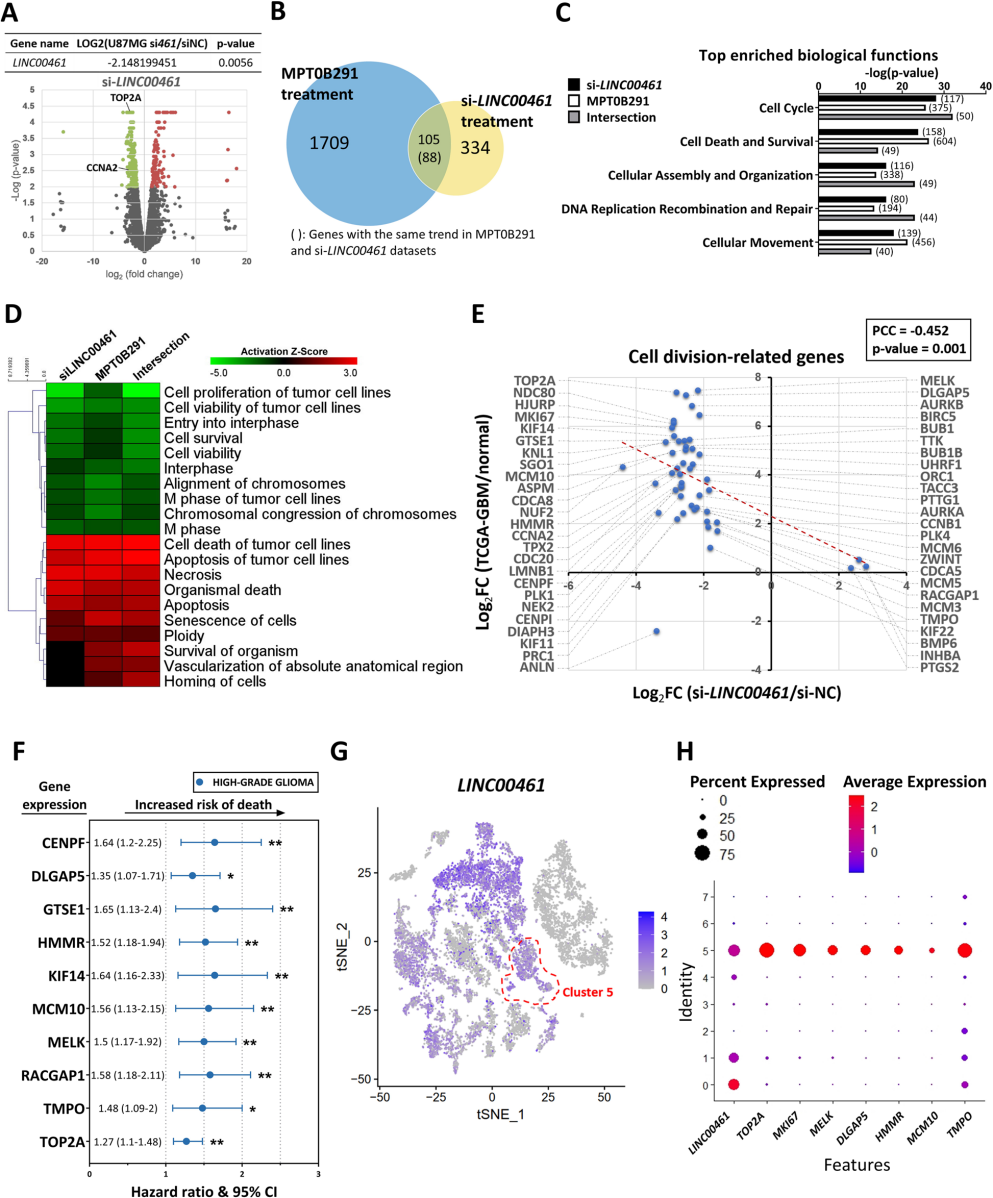
Involvement of *LINC00461* in HDAC6-mediated cell division and survival in GBM cells. **A** Volcano plot visualizing the differentially expressed genes between si-NC and si-*LINC00461* (si*461*) treatment. Green and red dots represent genes with significant downregulation and upregulation (*p*-value < 0.01), respectively, in si-*LINC00461* compared with si-NC. **B** Venn diagram illustrating overlaps between the number of genes that were significantly altered in MPT0B291-treated and si-*LINC00461*-treated cells. **C** Top five molecular and cellular functions of genes altered in si-*LINC00461*-treated (334 genes), MPT0B291-treated (1709 genes), and intersection subset (88 genes) by IPA. The number of genes involved in the biological process is shown on the right side of the bar chart. **D** Heatmap representation of the top 20 predicted influential biological functions after treatment by IPA. Positive activation Z-score (red) represents activated pathways, and negative activation Z-score (green) represents inhibited pathways. **E** Relationships between si-*LINC00461*-treated NGS data (in horizontal) and TCGA-GBM NGS data (in vertical) by Pearson’s correlation coefficient (PCC). Each dot represents the expression value of a cell-division-related gene that existed in the intersection subset. **F** Forest plots showing hazard ratios for risk of mortality in high-grade glioma patients with higher expressions of the indicated genes. Circles represent the hazard ratio, and bars on both sides denote their corresponding 95% confidence intervals (CI). The original survival curves were obtained from PROGgeneV2 (Fig. S4). Log-rank test. **G** Individual cells suspended/isolated directly from patient’s fresh GBM sample were used for single-cell RNA sequencing. Each dot denotes one cell with the expression of *LINC00461*, and the depth of blue represents the amount of *LINC00461* expression. The red dashed circle indicates cluster 5. **H** Expression profiles of *LINC00461* and cell-division-related genes in cell clusters from the single-cell RNA sequencing dataset. Dot size reflects the percent of cells expressing the gene, and dot color indicates average gene expression

To characterize the role of *LINC00461* in GBM cells, we performed a genome-wide analysis of gene expression in *LINC00461*-silenced U87MG cells, and identified that 115 genes were upregulated and 219 genes were downregulated (Fig. 5A). Interestingly, the 219 down-regulated genes included *TOP2A* and *CCNA2*, both containing a gene expressing cyclin A2 to maintain the G2/M transition phase of the cell cycle. A comprehensive analysis conducted by comparing the *LINC00461*-silenced dataset with the MPT0B291-treated dataset revealed 105 intersection genes (Fig. 5B). The expression of 88 genes showed the same trend in both treatments. Functional analysis by IPA revealed highly significant functional gene classes associated with “cell division (50 of 88 genes)” and “survival (49 of 88 genes)” (Fig. 5C). Furthermore, we examined the activity of several pathways using IPA, and found that either *LINC00461* depletion or MPT0B291 treatment significantly inhibited cell proliferation/survival and induced cell death/senescence in GBM (Fig. 5D). These data indicated that *LINC00461* is involved in HDAC6-regulated GBM malignancy. To further confirm the clinical relevance of the 50 cell-division-related genes appearing in the intersection dataset (88 genes), we analyzed the TCGA-GBM next-generation sequencing (NGS) datasets of 124 patients. A strong negative correlation was observed (PCC: − 0.452) (Fig. 5E). The result indicated that abnormalities in the expression of the 50 cell-division-related genes are inverted following the HDAC6/*LINC00461* inhibition in GBM. Moreover, survival analysis using clinical outcome data showed that several cell-division-related genes (*CENPF, DLGAP5, GTSE1, HMMR, KIF14, MCM10, MELK, RACGAP1, TMPO, and TOP2A*) have poor prognostic implications in high-grade gliomas (Fig. 5F and Fig. S4A), suggesting that the reducing expressions of these genes through HDAC6/LINC00461 inhibition may improve outcomes in GBM patients. Additionally, the importance of the HDAC6/*LINC00461*-mediated regulation in clinical GBM was analyzed using single-cell transcriptome sequencing datasets. Surprisingly, higher expressions of *LINC00461* and its associated downstream cell-division-related genes mentioned in Fig. 5E were enriched in a specific cluster (Cluster 5) of GBM cells (Fig. 5G–H). Consistently, functional analysis of the conserved markers in Cluster 5 by IPA indicated highly similar functional gene classes as that of the *LINC00461*-silenced and MPT0B291-treated datasets mentioned in Fig. 5C (Fig. S5A, Table S4).

### *LINC00461* depletion downregulates cell-cycle-related protein MELK by blocking the interaction between *LINC00461* and miR-485-3p

**Fig. 6:**
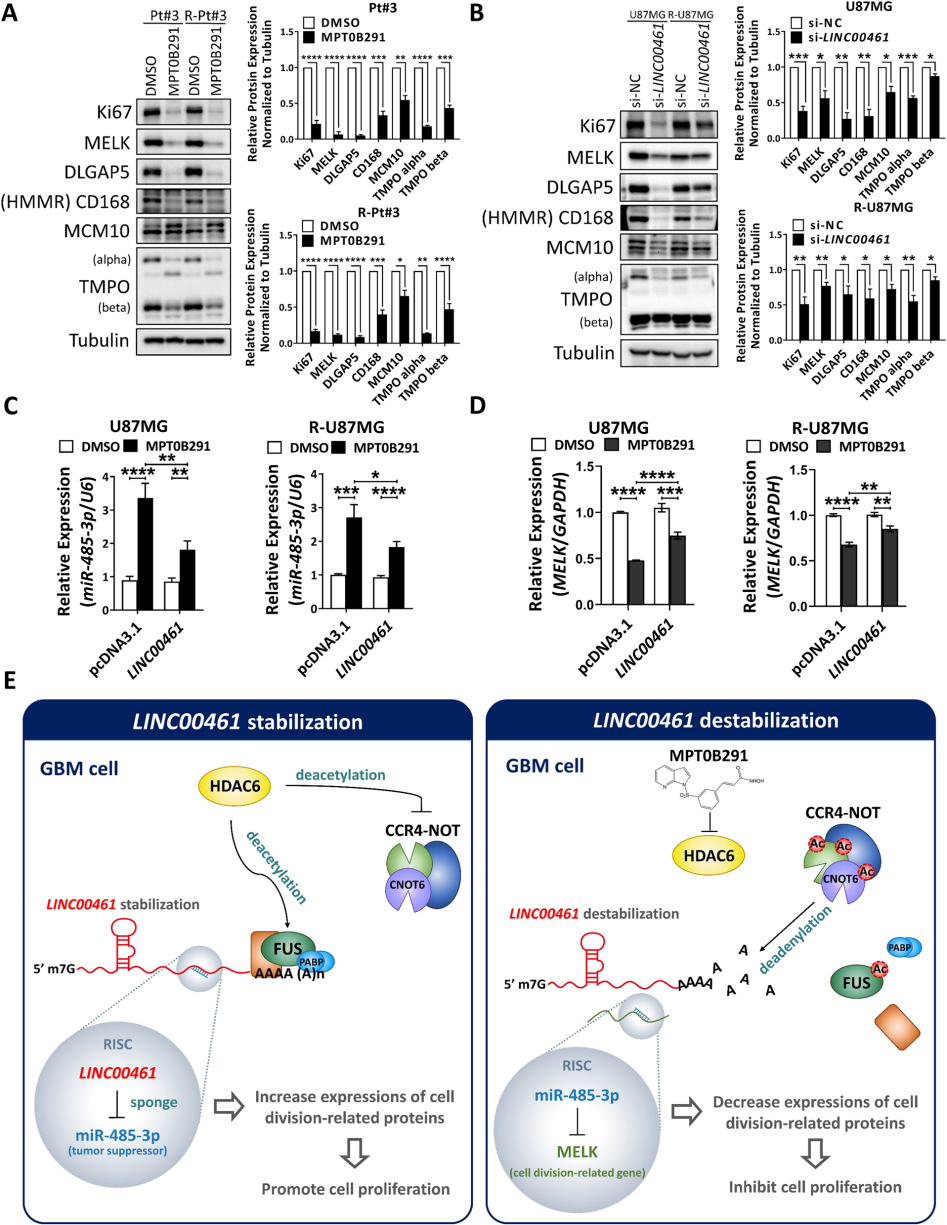
*LINC00461* maintains the expression of cell-cycle-related proteins by sponging tumor-suppressive miRNAs. **A** Effect of MPT0B291 on the protein expression of cell-division-related genes at 48 h in parental and TMZ-resistant Pt#3 cells. Quantification of expression from triplicate samples is shown alongside the data. Unpaired Student’s *t*-test. **B** Effect of *LINC00461* depletion on the protein expression levels of cell-division-related genes in parental and TMZ-resistant U87MG cells. Quantification of expression from triplicate samples is shown alongside the data. Unpaired Student’s *t*-test. **C** The combined effect of *LINC00461* elevation and MPT0B291 treatment on the expression of *miR-485-3p* in parental and TMZ-resistant U87MG cells. Unpaired Student’s *t*-test. **D** The combined effect of *LINC00461* elevation and MPT0B291 treatment on the expression of MELK in parental and TMZ-resistant U87MG cells. Unpaired Student’s *t*-test. **E** A schematic diagram illustrating how the HDAC6/RBP/*LINC00461* axis maintains the expression of cell-cycle-related protein MELK by sponging tumor-suppressive miR-485-3p, but the HDAC6 inhibition induced by MPT0B291 is able to block *LINC00461* functions, reducing GBM malignancy. The results are shown as mean ± SEM for triplicate samples in each group

We further investigated downstream targets of *LINC00461*, especially in cell-cycle-related proteins. Either MPT0B291 treatment or *LINC00461* depletion significantly downregulated the expression of several cell-division-regulated factors, including Ki67, maternal embryonic leucine zipper kinase (MELK), DLG associated protein 5 (DLGAP5), hyaluronan mediated motility receptor (HMMR), minichromosomal maintenance protein 10 (MCM10), and thymopoietin (TMPO) (Fig. 6A–B). Given that *LINC00461* is dominantly located in the cytoplasm [13], we explored its function as a miRNA sponge to repress the inhibitory effect of miRNAs on target mRNA. Prediction of the lncRNA-miRNA-mRNA networks (Fig. S6A) using three public databases (miRDB [24], miRWALK [25], and starBase [26]) revealed 13 intersecting miRNAs in the datasets (Fig. S6B). Of these, miR-485-3p, a tumor suppressor in GBM [27] was presumed to inhibit MCM10 and MELK. Thus, we examined whether *LINC00461* indeed played a role in sponging miR-485-3p. MPT0B291 treatment-mediated *LINC00461* inhibition led to significantly elevated miR-485-3p expression (Fig. 6C) but it significantly reduced MELK expression in parental and TMZ-resistant GBM cells (Fig. 6D). However, the simultaneous overexpression of *LINC00461* in cells decreased the induction of miR-485-3p levels (Fig. 6C) and reduction of MELK expression by MPT0B291 (Fig. 6D), suggesting that *LINC00461* maintains MELK expression by sponging miR-485-3p.

## Discussion

Although lncRNAs were considered non-functional and transcriptional noise initially, cumulative evidence over the past decade has unraveled that lncRNAs perform diverse biological functions [28]. Strategies for targeting lncRNAs in cancer treatment have gained widespread interest because they have vital roles in gene regulation and thus modulate hallmark processes of cancer [29]. Our previous studies demonstrated that HDAC inhibition using inhibitors represses stem-like properties in GBM that provide a potential therapeutic strategy to overcome drug resistance [10, 30]. Nevertheless, those two studies focused on HDAC-regulated protein-coding genes associated with drug resistance, but the role of HDACs on the impact of dysregulated lncRNAs remains unclear and hence needs further investigation. Here, we investigated the involvement of HDAC6 in *LINC00461* regulation by performing microarrays and RNA-seq analysis in stem-like and TMZ-resistant GBM cells with HDAC6 inhibition using MPT0B291 and siRNA.

According to a transition in investigative approaches from lncRNA annotation and molecular characterization to tumorigenic mouse models needed for deciphering the physiological roles of cancer-associated lncRNAs [29], we examined the impact of *LINC00461* on GBM growth and survival in vivo. Evidence gathered shows that MPT0B291 treatment significantly reduced subcutaneous tumor growth along with a decreased level of *LINC00461* and a negative correlation between *LINC00461* expression and survival of mice with intracerebral GBM, suggests a pivotal role for *LINC00461* as an oncogenic factor in GBM malignancy. Indeed, a previous study has demonstrated that the expression of *C130071C03Rik*, the mouse ortholog of *LINC00461*, is increased not only in precancerous conditions but also during glioma progression [13]. Some characteristics suggest that *LINC00461* is a potentially promising target for treating patients with GBM. First, previous literature on *LINC00461* demonstrated its oncogenic functions across diverse cancer types [13, 14, 31, 32]. Second, *LINC00461* displays a brain-specific expression pattern, which implies reduced unintended toxic effects associated with their targeting. Third, unlike most intergenic lncRNAs that show poor sequence conservation, *LINC00461* has a high degree of sequence homology across vertebrates [13], suggesting its essential role among such species [33].

The mechanisms underlying the regulation of mRNA stability have been extensively reviewed [34], whereas little is known about the modulation of lncRNA stability. Considering the inhibitory potency of MPT0B291 on HDAC1 and 2 (IC_50_ ≈ 0.31 and 1.16 μM, respectively) [10, 11], the impact of HDAC1 and HDAC2 on *LINC00461* expression was also examined in GBM cells using siRNA-mediated depletion. Interestingly, the silencing of neither HDAC1 nor HDAC2 was involved in modulating *LINC00461* expression (Fig. S7A), suggesting that a decrease in *LINC00461* expression by MPT0B291 is mainly due to the inhibition of HDAC6 rather than that of HDAC1 or HDAC2. Although several aspects discriminate lncRNAs from protein-coding mRNA, they also share certain similarities [35]. Numerous lncRNAs are subjected to transcriptional editing, for example, splicing, polyadenylation, and 5′ capping, just like mRNAs [35]. Post-transcriptional regulation of lncRNAs is mediated by RBPs in mainly two aspects: stability and localization. Our results identified two HDAC6-associated proteins (CNOT6 and FUS) that might explain a shorter half-life of *LINC00461* in response to MPT0B291 treatment (Fig. S7B). Because the enzyme activity of CNOT6 is required for deadenylation by the CCR4-NOT nuclease complex [36], and acetylation enhances CNOT6 activity inducing RNA degradation [22], we predict that high levels of HDAC6 in GBM cells inhibit CNOT6 acetylation, leading to *LINC00461* stabilization, while MPT0B291 treatment induces CNOT6 acetylation, promoting *LINC00461* degradation (Fig. S7B). FUS-containing 3′-end processing machinery facilitates mRNA stability [23], and lysine status regulated by cyclic adenosine monophosphate–response element binding protein/p300 and histone deacetylase families contribute to FUS function [37]. Therefore, in GBM cells, high levels of HDAC6 may increase the RNA-binding affinity of FUS, resulting in *LINC00461* stabilization, whereas MPT0B291 treatment induces FUS acetylation, promoting *LINC00461* degradation (Fig. S7B). Although our current results prove that HDAC6 inhibition increases CNOT6 and decreases FUS protein levels, further studies are needed to confirm whether HDAC6 directly regulates the lysine status of these two proteins.

Although recent studies have shown the oncogenic roles of *LINC00461* in tumors [13, 14, 31, 32], the molecular mechanisms underlying *LINC00461* regulation remain unclear. To understand the function of *LINC00461* comprehensively, we compared the *LINC00461*-silenced dataset with the clinical TCGA-GBM dataset. Pathway enrichment analysis of intersected genes in these two datasets revealed cell growth, which is consistent with the findings that *LINC00461* promoted GBM proliferation through the regulation of TOP2A expression. TOP2A is a nuclear enzyme that controls and alters the topologic states of DNA during transcription and mitosis, facilitating gene expression and mitotic progression, respectively, in tumor cells [38]. Here, we found that the HDAC6/*LINC00461* axis inhibition downregulated TOP2A expression in parental and TMZ-resistant cells, thereby explaining the perturbation of cell-cycle progression into G2/M phase. In addition, a previous study has demonstrated that *LINC00461* knockdown reduces expressions of a few cell-division-related genes, including cyclin D1, CDK4, cyclin A2, and cyclin E in U87MG [39]. Although genome-wide analysis with critical criteria for selecting potential *LINC00461* downstream targets from altered genes in the *LINC00461*-silencing dataset only identified cyclin A [39] and TOP2A [13] as presented previously, we measured the expressions of the other cell-division-related genes mentioned above in our GBM cell model. Significant decreases in the expression levels of these cell-division-related genes (cyclin D1, CDK4, cyclin A2, and cyclin E1) following *LINC00461* knockdown were observed in parental and TMZ-resistant U87MG (Fig. S8A). Furthermore, *LINC00461* knockdown significantly downregulated protein expressions of cyclin D1 and CDK1 (Fig. S8B), which is consistent with the findings of a previous report [39]. Except for the *LINC00461*-regulated cell-cycle-related genes, which have already been proved, numerous cell-division-related genes that are modulated by *LINC00461* were revealed in this study for the first time (Figs. 5E and 6). In summary, *LINC00461* displays a widespread regulation of cell-division-related proteins that are indispensable for GBM growth.

Apart from exploring the function of *LINC00461* using the public clinical dataset, we also performed transcriptome profiling of clinical samples at the single-cell level. Recent advances in the development of NGS technologies have overcome conventional profiling methods that assess bulk populations and provide the opportunity to gain insights into the characterization of individual cells [40]. Considering the heterogeneity of tumor biology, we explored whether there is a cluster of cells overexpressing *LINC00461*. Surprisingly, our findings revealed that the cells in Cluster 5 showed highly elevated expressions of *LINC00461* and cell-division-related genes simultaneously and also displayed biomarkers enriched in pathways associated with cell division (Fig. S5A, Table S1). Therefore, the cell characteristics in Cluster 5 are require further study to clarify the clinical malignancy of GBM and the development of treatment strategies.

Like proteins, appropriate subcellular localization patterns of lncRNAs allow for the primary determination of their molecular functions [41]. Our observations in xenograft tumors through both RNA-ISH and RNA-FISH staining demonstrated that *LINC00461* localizes to the cytoplasm rather than to the nucleus. Cytoplasmic lncRNAs modulate mRNA fate by acting as miRNA sponges that compete for miRNA binding [42]. To dissect the regulatory mechanism between *LINC00461* and cell-division-related proteins comprehensively, we used three reliable public datasets to predict lncRNA-miRNA-mRNA networks. Downregulation of MCM10 and MELK proteins by *LINC00461* depletion combined with the induction of miR-485-3p by *LINC00461* inhibition using MPT0B291 suggests that this strategy is effective for identifying the downstream regulatory networks of *LINC00461*.

## Conclusions

Recent research has unraveled the indispensable roles of lncRNAs in regulating GBM malignancy, including cancer stem-like features [8]. Nevertheless, the mechanism by which cancer-associated lncRNAs are dysregulated remains unexplored. Here, our results provide an insight into lncRNA regulation by HDAC proteins and lead to a comprehensive understanding of *LINC00461*-associated networks in GBM malignancy, by expounding the utility of the HDAC6/RBPs/*LINC00461* axis as a potential therapeutic approach for treating patients with GBM (Fig. 6E). However, this research has thrown up many questions that need further investigation. Future research should focus on the identification of the lysine residue of RBPs that is deacetylated by HDAC6, isolating the *LINC00461*-miRNA-mRNA axis that is the most important compared to the others, and understanding the mechanism of targeting *LINC00461*-overexpressed cells effectively in clinical practice.

## Supplementary Information

**Additional file 1: Figure S1.** MPT0B291 commonly alters two lncRNAs (*LINC00461* and *LINC01559*) in stem-like and TMZ-resistant U87MG cells. Volcano plots show overlapping lncRNAs between two microarray data (MS1040791 and MS1060987). Relative expression level (log2) to control (DMSO) of each lncRNA is shown in the x-axis. y-axis indicates SNR (signal-to-noise ratio) value (log2) of each lncRNA. Genes with significant upregulation (red) and downregulation (green) in both microarray datasets are highlighted. **Figure S2.** MPT0B291 decreases Ki-67 expression in vivo. (A) IHC detected the protein expressions of proliferation (Ki-67) and apoptosis (cleaved caspase-3) marker. Serial sections of control and MPT0B291-treated xenografts were hybridized with either an antibody against Ki-67 (upper two panels) or cleaved caspases-3 (lower two panels), and cell nuclei were stained with hematoxylin. Scale bars indicate 20 μm and 200 μm. Mean intensity of Ki-67 and cleaved caspases-3 staining in the cells of control and MPT0B291-treated xenografts were semi-quantitative determined. Unpaired Student’s *t*-test. **Figure S3.** Interaction between HDAC6 and RNA-binding proteins (CNOT6 and FUS). (A) Flag-tagged CNOT6-expressed U87MG cells were treated with 10 μM MPT0B291, 10 μM Trichostatin A (TSA), or DMSO for 2 h. Flag-CNOT6 protein was immunoprecipitated and the precipitated samples were then analyzed by immunoblotting analysis with antibodies of HDAC6 or Flag-M2. (B) Enrichment of HuR from the lysate by desthiobiotin-labeled androgen receptor (AR) 3′-UTR validates the efficiency of RNA-protein pull-down assay (left panel). Protein lystaes from TMZ-resistant Pt#3 cells were also used for the RNA-protein pull-down assay with desthiobiotin-labeled *LINC00461*, and both poly(A)-binding protein (PABP) and HuR were slightly detected in the pull-down sample, but desthiobiotin-labeled *LINC00461* did not bind HDAC6 directly. (L = lysate; FT1 = flow-through in first wash; FT2 = flow-through in second wash; E = eluate). (C) Quantification of CNOT6 and FUS protein expression from the parental and TMZ-resistant Pt#3 cells either treated with MPT0B291 or si-HDAC6. Unpaired Student’s t-test. The results are shown as mean ± SEM for triplicate samples in each group. **Figure S4.** Cell division-related genes show poor prognostic implications in high-grade glioma patients. (A) Kaplan–Meier curves compare the survival outcomes in high-grade glioma patients with high (red) and low (green) expressions of cell division-related genes. All survival curves were obtained from PROGgeneV2. Log-rank test. **Figure S5.** Functional analysis of the conserved markers in cluster 5 from scRNA-seq data reveals gene classes associated with cell division and survival. Core analysis using IPA revealed the top ten molecular and cellular functions of highly conserved marker genes in cluster 5 of patient-derived GBM cells. Log(*p*-value) indicates the significance of enrichment for highly expressed marker genes from our dataset. The threshold for significance was set at a *p*-value < 0.05. **Figure S6.** Three datasets identify potential interaction networks between 13 miRNAs and cell division-related genes. (A) Pipeline for the identification of potential regulatory *LINC00461*-miRNA-mRNA networks. (B) The miRNA-mRNA networks were identified using three public databases (miRDB, miRWALK, and starBase). **Figure S7.** The regulation of *LINC00461* stability. Protein expression levels of HDAC1 and HDAC2 in parental and TMZ-resistant U87MG cells treated with either si-NC or si-HDAC1/2. Effect of either HDAC1 or HDAC2 depletion on *LINC00461* expression in parental and TMZ-resistant U87MG cells. n.s., not significant, unpaired Student’s t-test. (B) A schematic diagram illustrates the proposed regulatory mechanism underlying HDAC6 controls the *LINC00461* stability via regulating both the RNA-binding activity of FUS (fused in sarcoma)/PABP and the activity of deadenylases of human Ccr4-Not complex. **Figure S8.***LINC00461* knockdown downregulates the expressions of cell division-related molecules. (A) Expressions of the cell division-related mRNAs (cyclin D1, CDK4, cyclin A2, cyclin E1, MELK, and MCM10) in parental and TMZ-resistant R-U87MG cells that were treated with either si-NC or si-*LINC00461*. Unpaired Student’s t-test. (B) Effect of *LINC00461* depletion on the protein expression levels of cell division-related molecules (cyclin D1 and CDK1) in parental and TMZ-resistant U87MG cells. **Table S1.** siRNA targeted-sequences for each gene. **Table S2.** The primer or probe sequences for each gene used in SYBR green and TaqMan qPCR assays. **Table S3.** Custom LNA™ detection probes sequences. **Table S4.** List of biomarkers with fold-change more significant than 1.5 in cluster 5.

## Data Availability

All data supporting the findings of this study are available within the article and supplementary data. The RNA-seq data discussed in this publication have been deposited in NCBI’s Gene Expression Omnibus (GEO) and are accessible through GEO Series accession number GSE182220 (https://www.ncbi.nlm.nih.gov/geo/query/acc.cgi?acc=GSE182220).

## Abbreviations

ActD: Actinomycin D
AP: Alkaline phosphatase
AR: Androgen receptor
CNOT6: CCR4-NOT core exoribonuclease subunit 6
CI: Confidence interval
DIG: Digoxigenin
DLGAP5: DLG associated protein 5
ECL: Enhanced chemiluminescence
FISH: Fluorescence in situ hybridization
FUS: Fused in sarcoma
GBM: Glioblastoma
GEO: Gene expression omnibus
HDAC: Histone deacetylase
HMMR: Hyaluronan mediated motility receptor
IPA: Ingenuity pathway analysis
ISH: In situ hybridization
ITGB3: Integrin β3
lncRNAs: Long non-coding RNAs
iTRAQ: Isobaric tags for relative and absolute quantitation
MCM10: Minichromosomal maintenance protein 10
MELK: Maternal embryonic leucine zipper kinase
MPT0B291: Azaindolylsulfonamide compound 12
MTT: 3-(4,5-dimethylthiazol-2-yl)-2,5-diphenyltetrazolium bromide
NGS: Next-generation sequencing
PCC: Pearson’s correlation coefficient
RBPs: RNA-binding proteins
scRNA-seq: Single-cell RNA sequencing
SNR: Signal-to-noise-ratio
TCGA: The cancer genome atlas
TMPO: Thymopoietin
TMZ: Temozolomide
TOP2A: Topoisomerase II alpha
TRITC: Tetramethylrhodamine-isothiocyanate
TSA: Trichostatin A
