# Supplementary material for: HDAC6 involves in regulating the lncRNA-microRNA-mRNA network to promote the proliferation of glioblastoma cells
Source: J Exp Clin Cancer Res. 2022 Feb 2;41:47. doi: 10.1186/s13046-022-02257-w (PMC8809020; doi:10.1186/s13046-022-02257-w)
Supplement: Supplementary file 1 — Additional file 1: Figure S1. MPT0B291 commonly alters two lncRNAs (LINC00461 and LINC01559) in stem-like and TMZ-resistant U87MG cells. Volcano plots show overlapping lncRNAs between two microarray data (MS1040791 and MS1060987). Relative expression level (log2) to control (DMSO) of each lncRNA is shown in the x-axis. y-axis indicates SNR (signal-to-noise ratio) value (log2) of each lncRNA. Genes with significant upregulation (red) and downregulation (green) in both microarray datasets are highlighted. Figure S2. MPT0B291 decreases Ki-67 expression in vivo. (A) IHC detected the protein expressions of proliferation (Ki-67) and apoptosis (cleaved caspase-3) marker. Serial sections of control and MPT0B291-treated xenografts were hybridized with either an antibody against Ki-67 (upper two panels) or cleaved caspases-3 (lower two panels), and cell nuclei were stained with hematoxylin. Scale bars indicate 20 μm and 200 μm. Mean intensity of Ki-67 and cleaved caspases-3 staining in the cells of control and MPT0B291-treated xenografts were semi-quantitative determined. Unpaired Student’s t-test. Figure S3. Interaction between HDAC6 and RNA-binding proteins (CNOT6 and FUS). (A) Flag-tagged CNOT6-expressed U87MG cells were treated with 10 μM MPT0B291, 10 μM Trichostatin A (TSA), or DMSO for 2 h. Flag-CNOT6 protein was immunoprecipitated and the precipitated samples were then analyzed by immunoblotting analysis with antibodies of HDAC6 or Flag-M2. (B) Enrichment of HuR from the lysate by desthiobiotin-labeled androgen receptor (AR) 3′-UTR validates the efficiency of RNA-protein pull-down assay (left panel). Protein lystaes from TMZ-resistant Pt#3 cells were also used for the RNA-protein pull-down assay with desthiobiotin-labeled LINC00461, and both poly(A)-binding protein (PABP) and HuR were slightly detected in the pull-down sample, but desthiobiotin-labeled LINC00461 did not bind HDAC6 directly. (L = lysate; FT1 = flow-through in first wash; FT2 = flow-through in second was [file 13046_2022_2257_MOESM1_ESM.docx]

**Supplementary figures and legends**

**Figure S1**


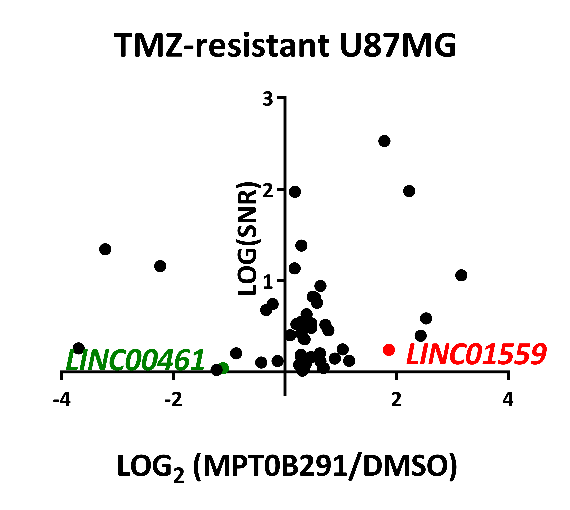


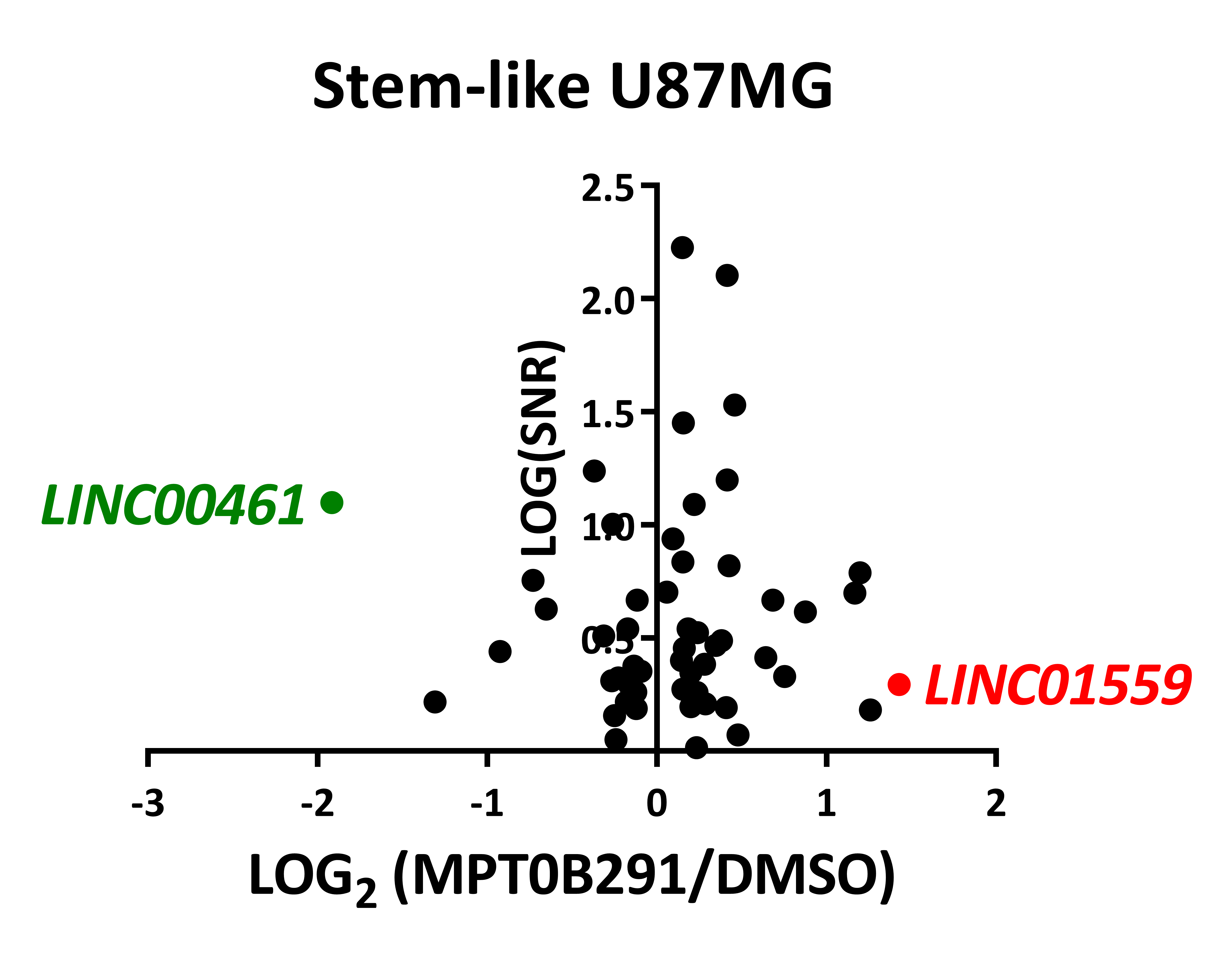


**Figure S1.** MPT0B291 commonly alters two lncRNAs (*LINC00461* and *LINC01559*) in stem-like and TMZ-resistant U87MG cells. Volcano plots show overlapping lncRNAs between two microarray data (MS1040791 and MS1060987). Relative expression level (log2) to control (DMSO) of each lncRNA is shown in the x-axis. y-axis indicates SNR (signal-to-noise ratio) value (log2) of each lncRNA. Genes with significant upregulation (red) and downregulation (green) in both microarray datasets are highlighted.

**Figure S2**


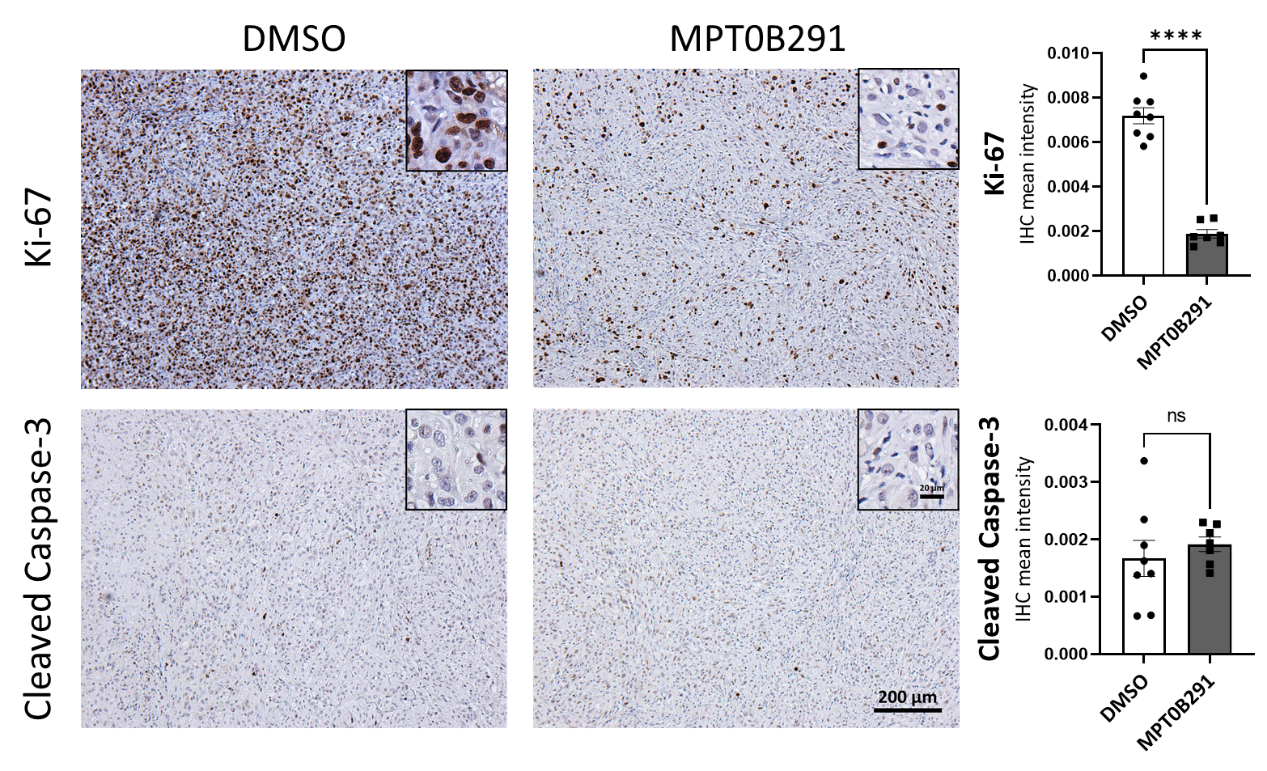


**Figure S2.** MPT0B291 decreases Ki-67 expression *in vivo*. (A) IHC detected the protein expressions of proliferation (Ki-67) and apoptosis (cleaved caspase-3) marker. Serial sections of control and MPT0B291-treated xenografts were hybridized with either an antibody against Ki-67 (upper two panels) or cleaved caspases-3 (lower two panels), and cell nuclei were stained with hematoxylin. Scale bars indicate 20 µm and 200 µm. Mean intensity of Ki-67 and cleaved caspases-3 staining in the cells of control and MPT0B291-treated xenografts were semi-quantitative determined. Unpaired Student’s *t*-test.

**Figure S3**


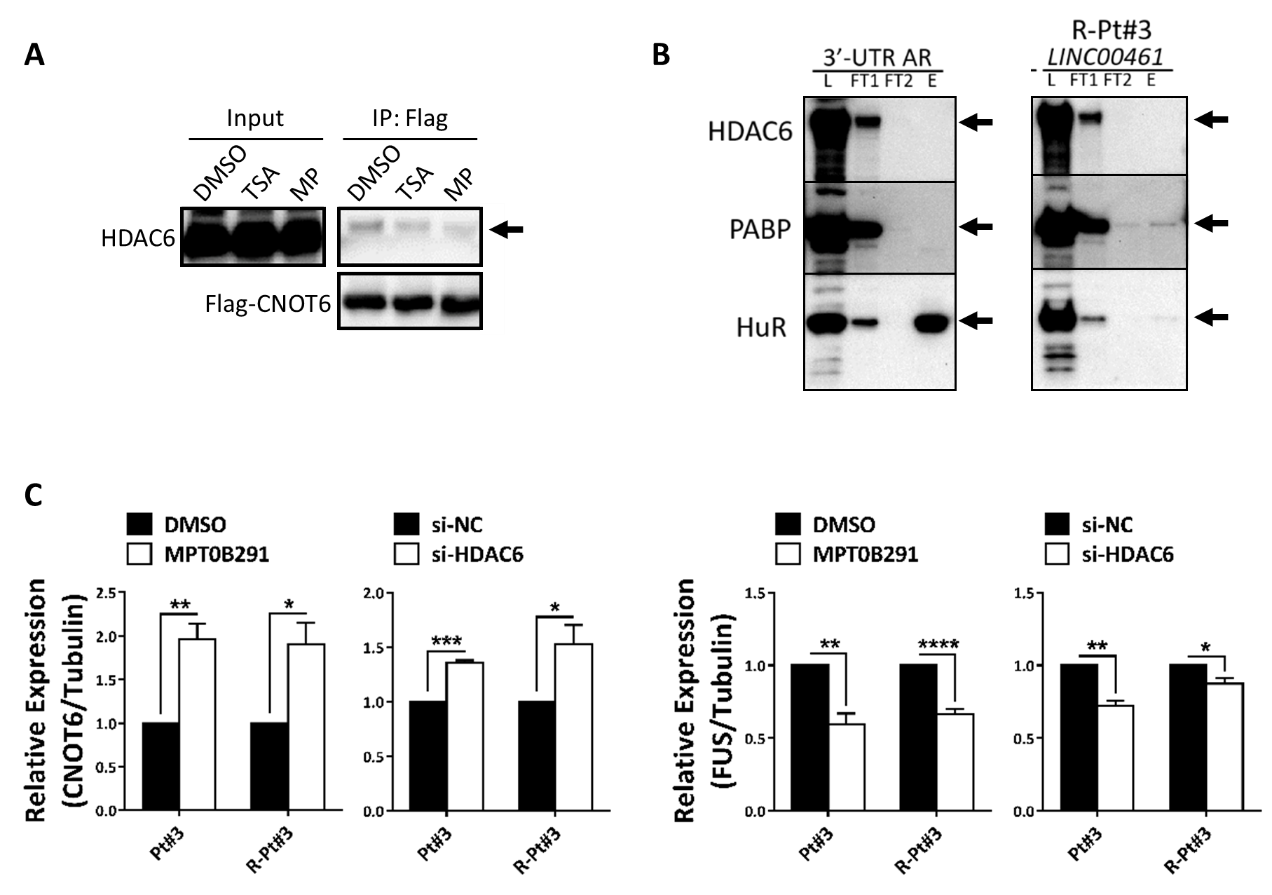


**Figure S3.** Interaction between HDAC6 and RNA-binding proteins (CNOT6 and FUS). (A) Flag-tagged CNOT6-expressed U87MG cells were treated with 10 µM MPT0B291, 10 µM Trichostatin A (TSA), or DMSO for 2 h. Flag-CNOT6 protein was immunoprecipitated and the precipitated samples were then analyzed by immunoblotting analysis with antibodies of HDAC6 or Flag-M2. (B) Enrichment of HuR from the lysate by desthiobiotin-labeled androgen receptor (AR) 3´-UTR validates the efficiency of RNA-protein pull-down assay (left panel). Protein lystaes from TMZ-resistant Pt#3 cells were also used for the RNA-protein pull-down assay with desthiobiotin-labeled *LINC00461*, and both poly(A)-binding protein (PABP) and HuR were slightly detected in the pull-down sample, but desthiobiotin-labeled *LINC00461* did not bind HDAC6 directly. (L = lysate; FT1 = flow-through in first wash; FT2 = flow-through in second wash; E = eluate). (C) Quantification of CNOT6 and FUS protein expression from the parental and TMZ-resistant Pt#3 cells either treated with MPT0B291 or si-HDAC6. Unpaired Student’s t-test. The results are shown as mean ± SEM for triplicate samples in each group.

**Figure S4**

**
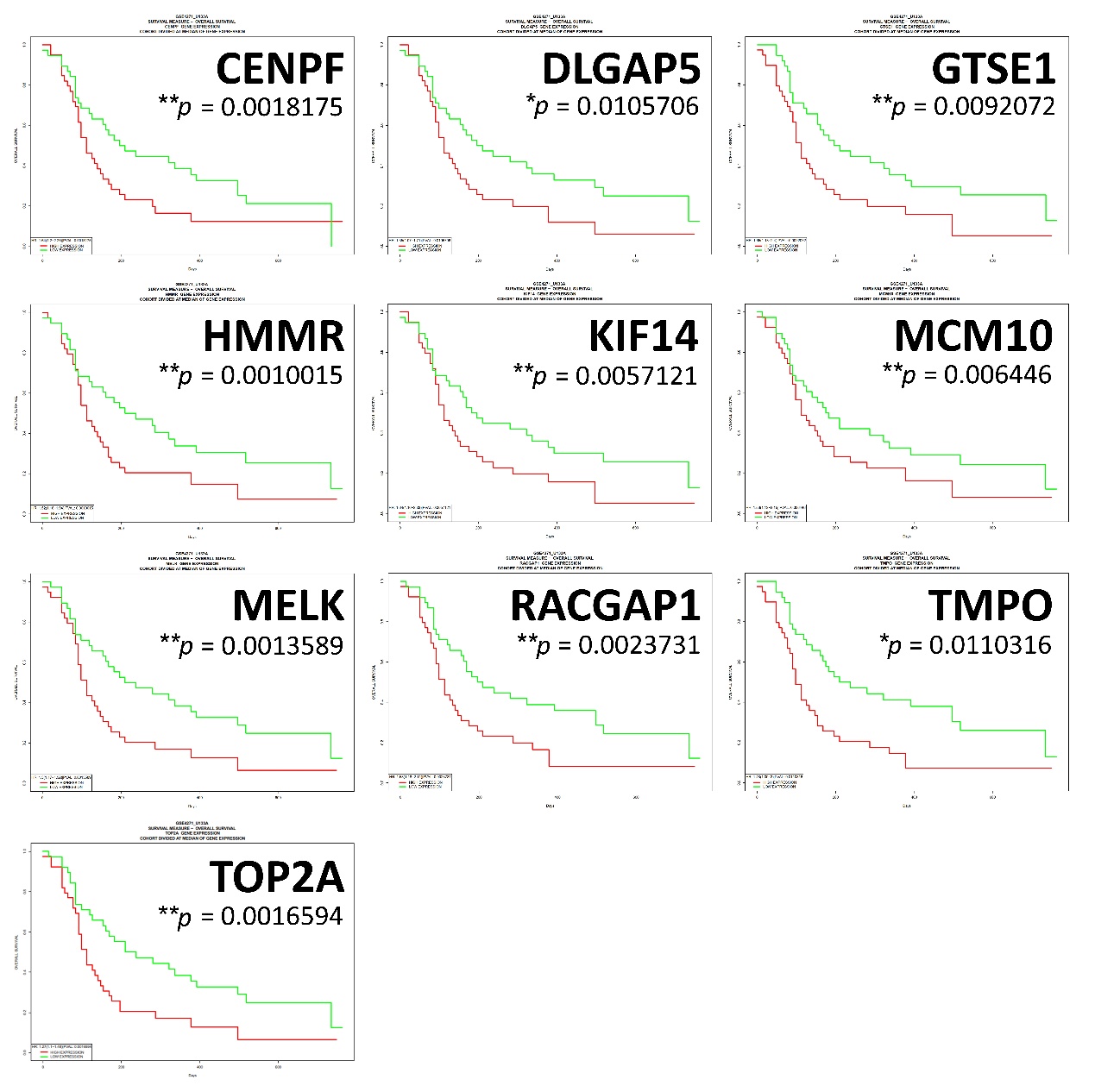
**

**Figure S4.** Cell division-related genes show poor prognostic implications in high-grade glioma patients. (A) Kaplan–Meier curves compare the survival outcomes in high-grade glioma patients with high (red) and low (green) expressions of cell division-related genes. All survival curves were obtained from PROGgeneV2. Log-rank test.

**Figure S5**


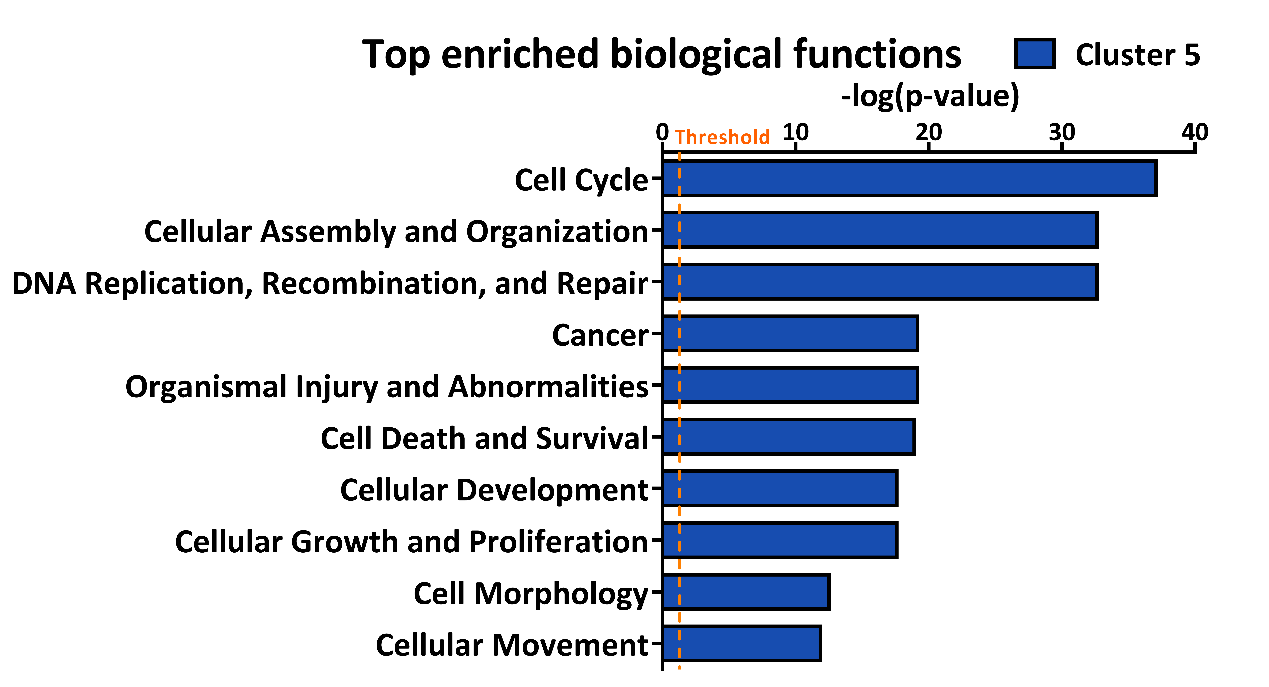


**Figure S5.** Functional analysis of the conserved markers in cluster 5 from scRNA-seq data reveals gene classes associated with cell division and survival. Core analysis using IPA revealed the top ten molecular and cellular functions of highly conserved marker genes in cluster 5 of patient-derived GBM cells. Log(p-value) indicates the significance of enrichment for highly expressed marker genes from our dataset. The threshold for significance was set at a p-value < 0.05.

**Figure S6**

**A**

**
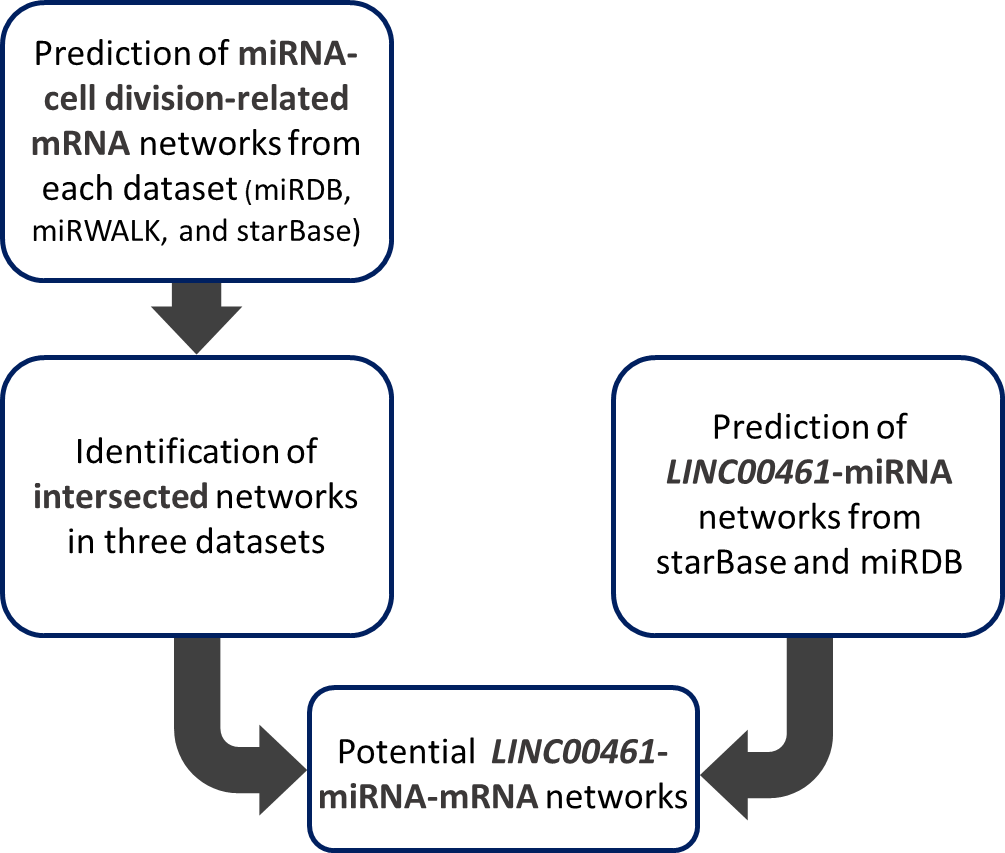
**

**B
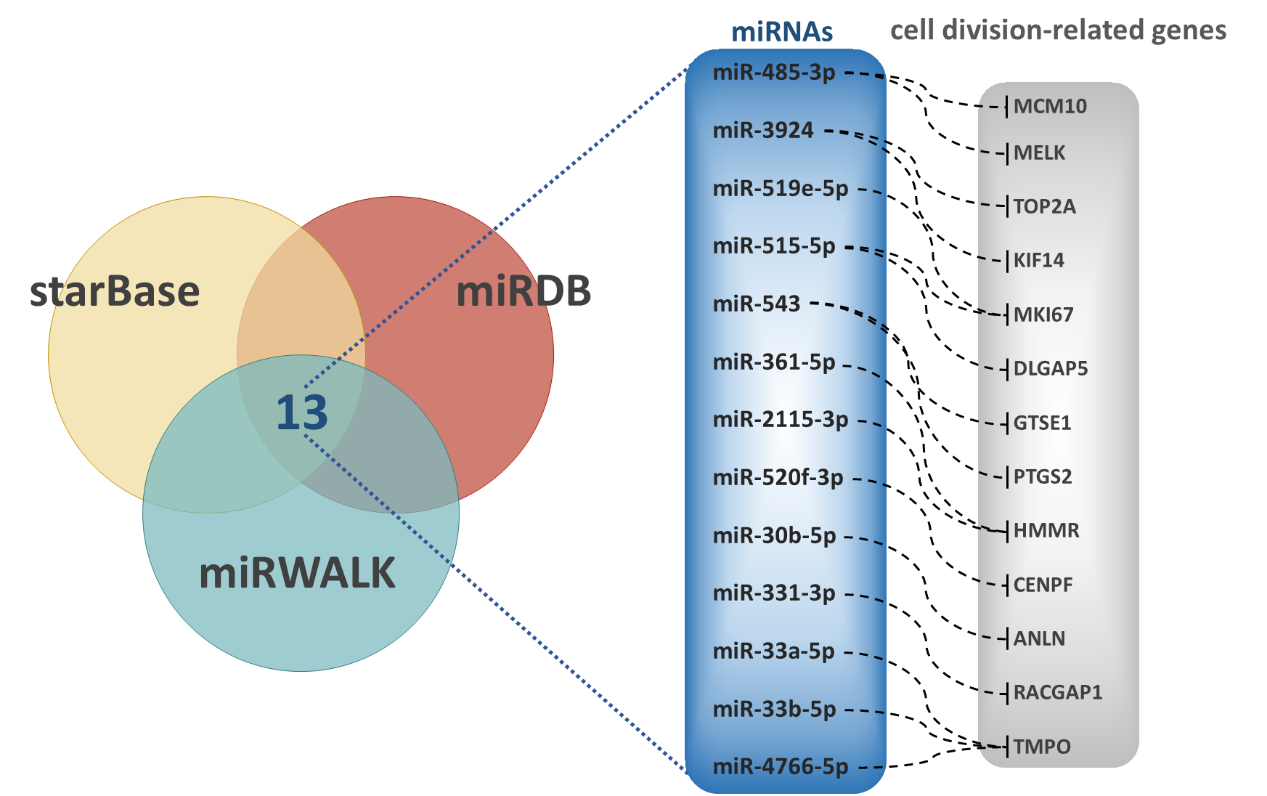
**

**Figure S6**. Three datasets identify potential interaction networks between 13 miRNAs and cell division-related genes. (A) Pipeline for the identification of potential regulatory *LINC00461*-miRNA-mRNA networks. (B) The miRNA-mRNA networks were identified using three public databases (miRDB, miRWALK, and starBase).

**Figure S7**


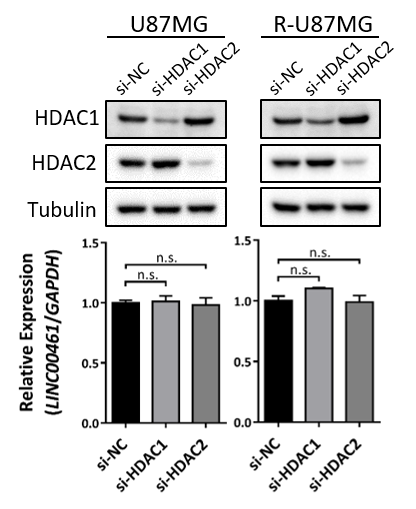
**
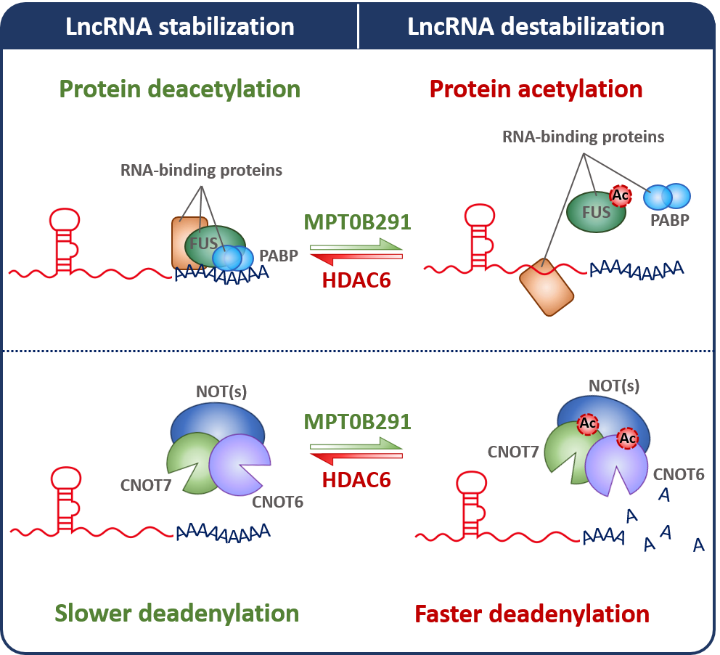
A B**

**Figure S7.** The regulation of *LINC00461* stability. Protein expression levels of HDAC1 and HDAC2 in parental and TMZ-resistant U87MG cells treated with either si-NC or si-HDAC1/2. Effect of either HDAC1 or HDAC2 depletion on *LINC00461* expression in parental and TMZ-resistant U87MG cells. n.s., not significant, unpaired Student’s t-test. (B) A schematic diagram illustrates the proposed regulatory mechanism underlying HDAC6 controls the *LINC00461* stability via regulating both the RNA-binding activity of FUS (fused in sarcoma)/PABP and the activity of deadenylases of human Ccr4-Not complex.

**Figure S8**

**A**

**
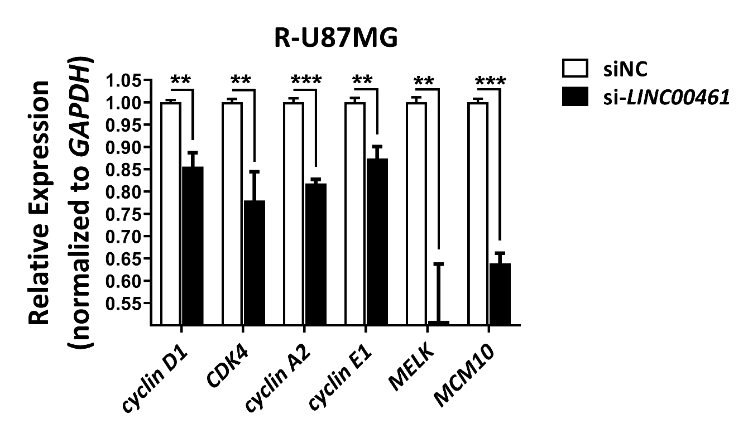

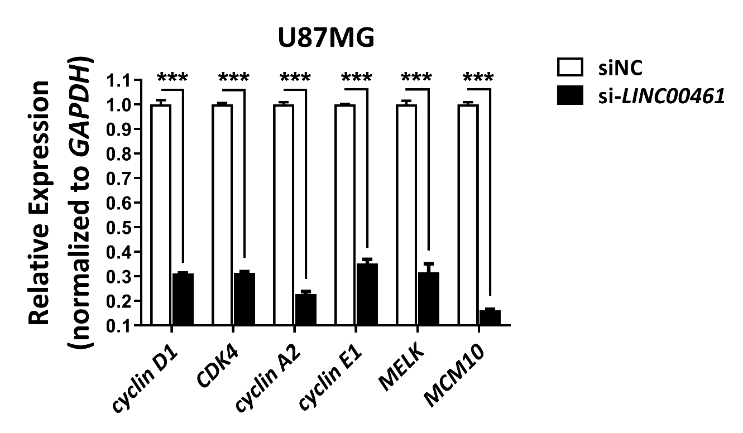
**


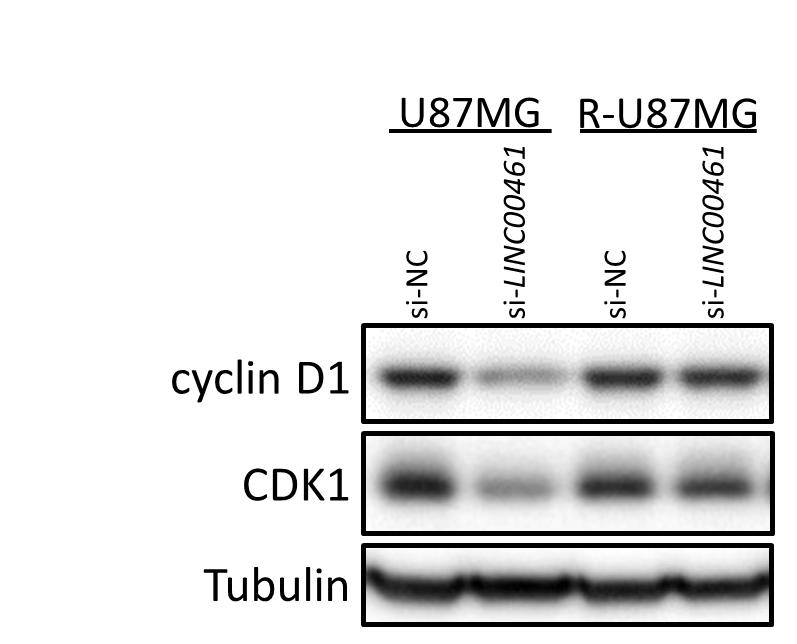


**B**

**Figure S8.** *LINC00461* knockdown downregulates the expressions of cell division-related molecules. (A) Expressions of the cell division-related mRNAs (cyclin D1, CDK4, cyclin A2, cyclin E1, MELK, and MCM10) in parental and TMZ-resistant R-U87MG cells that were treated with either si-NC or si-*LINC00461*. Unpaired Student’s t-test. (B) Effect of *LINC00461* depletion on the protein expression levels of cell division-related molecules (cyclin D1 and CDK1) in parental and TMZ-resistant U87MG cells.

**Supplementary tables**

**Table S1.** siRNA targeted-sequences for each gene

| Target gene | Targeted sequences (5' to 3') |
| --- | --- |
| *Sp1* | GCCAAUAGCUACUCAACUA |
|  | GAAGGGAGGCCCAGGUGUA |
|  | GGGCAGACCUUUACAACUC |
|  | CUACAGAGGCACAAACGUA |
| *HDAC1* | ACUAUGGUCUCUACCGAAA |
|  | GCAAGUAUUAUGCUGUUAA |
|  | CCGGUCAUGUCCAAAGUAA |
|  | CCACAGCGAUGACUACAUU |
| *HDAC2* | GCGGAUAGCUUGUGAUGAA |
|  | GCAAAGAAAGCUAGAAUUG |
|  | GAUAACAUGUCUGAGUAUA |
|  | GAUCGUGUAAUGACGGUAU |
| *HDAC6* | GGGAGGUUCUUGUGAGAUC |
|  | GGAGGGUCCUUAUCGUAGA |
|  | GCAGUUAAAUGAAUUCCAU |
|  | GUUCACAGCCUAGAAUAUA |
| *FUS* | GAUCAAUCCUCCAUGAGUA |
|  | GGACAGCAGAGUUACAGUG |
|  | GGACAGCAGCAAAGCUAUA |
|  | GAGCAGCUAUUCUUCUUAU |
| *LINC00461* | AAGAAAAGUUCGGAAUAAACUCUUU |
| NON-TARGET | UGGUUUACAUGUCGACUAA |
|  | UGGUUUACAUGUUGUGUGA |
|  | UGGUUUACAUGUUUUCUGA |
|  | UGGUUUACAUGUUUUCCUA |

**Table S2.** The primer or probe sequences for each gene used in SYBR green and TaqMan qPCR assays

Oligonucleotides used for SYBR Green qPCR assays

| Target gene | Targeted sequences (5’ to 3’) |
| --- | --- |
| *GAPDH* | Forward: CCATCACCATCTTCCAGGAG |
|  | Reverse: CCTGCTTCACCACCTTCTTG |
| *LINC00461* | Forward: GACCGTTGAGAAGACCCTTG |
|  | Reverse: TGCCTTAATTGAACCTCCTTG |
| *TOP2A* | Forward: AATCTCAGAGCTTCCCGTCA |
|  | Reverse: TGCCTCTGCCAGTTTTTCTT |
| *cyclin D1* | Forward: ATCAAGTGTGACCCGGACTG |
|  | Reverse: CTTGGGGTCCATGTTCTGCT |
| *CDK4* | Forward: TGTGGCCCTCAAGAGTGTGA |
|  | Reverse: ACGTCCATCAGCCGGACAAC |
| *cyclin E1* | Forward: GCCAGCCTTGGGACAATAATG |
|  | Reverse: CTTGCACGTTGAGTTTGGGT |
| *cyclin A2* | Forward: AAGACGAGACGGGTTGCAC |
|  | Reverse: CATGAATGGTGAACGCAGGC |
| *MELK* | Forward: AAGGTCAAACTTGCCTGCCA |
|  | Reverse: CTCCTCCAGGGCAGTACTCA |
| *MCM10* | Forward: GCCCAAAACCAGCCATCAAG |
|  | Reverse: TGAGCTCTGCAGAAATCGCT |

Oligonucleotides used for TaqMan qPCR miRNA assays

| Target gene | Targeted sequences (5' to 3') | |
| --- | --- | --- |
| *RNU6-1* | RT prmers | ATGGAACGCTTCACGAATTTG |
| *miR-485-3p* |  | GTCGTATCCAGTGCAGGGTCCGAGGTATTCGCACTG  GATACGACAGAGAG |
| *RNU6-1* | qPCR primers | Forward: CTCGCTTCGGCAGCACATAT |
|  |  | Reverse: ATGGAACGCTTCACGAATTTG |
| *miR-485-3p* |  | Forward: CGCGGTCATACACGGCT |
|  |  | Reverse: AGTGCAGGGTCCGAGGTATT |
| *RNU6-1* | qPCR probes | ACGATACAGAGAAGAT |
| *miR-485-3p* |  | ACGACAGAGAGGAGAGC |

**Table S3. Custom LNA^TM^ detection probes sequences**

| **Target gene** | **Targeted sequences (5' to 3')** |
| --- | --- |
| **Scramble** | **GTGTAACACGTCTATACGCCCA/3DiG_N/** |
| ***LINC00461*** | **TAGGTACCAGACATCTCTGCAA/3DiG_N/** |

**Table S4.** List of biomarkers with fold-change more significant than 1.5 in cluster 5

| Gene ID | avg_logFC | pct.1 | pct.2 | p_val_adj |
| --- | --- | --- | --- | --- |
| CENPF | 2.409561595 | 0.995 | 0.176 | 0 |
| TOP2A | 2.196906473 | 0.989 | 0.117 | 0 |
| NUSAP1 | 2.037857102 | 0.983 | 0.127 | 0 |
| YEATS4 | 1.997595964 | 0.969 | 0.388 | 0 |
| HMGB2 | 1.812968014 | 0.996 | 0.47 | 0 |
| HIST1H1D | 1.757697542 | 0.79 | 0.113 | 0 |
| ASPM | 1.736764836 | 0.829 | 0.074 | 0 |
| PTTG1 | 1.527093363 | 0.901 | 0.182 | 0 |
| UBE2C | 1.524868577 | 0.924 | 0.095 | 0 |
| UBE2T | 1.488755221 | 0.917 | 0.158 | 0 |
| HIST1H1B | 1.457016193 | 0.785 | 0.068 | 0 |
| CKAP2 | 1.442940934 | 0.935 | 0.247 | 0 |
| PCLAF | 1.436010078 | 0.877 | 0.142 | 0 |
| PRC1 | 1.427334736 | 0.893 | 0.103 | 0 |
| TPX2 | 1.35438473 | 0.895 | 0.087 | 0 |
| HIST1H3B | 1.291395987 | 0.733 | 0.054 | 0 |
| HIST1H3D | 1.284202153 | 0.729 | 0.068 | 0 |
| RRM2 | 1.273839982 | 0.845 | 0.079 | 0 |
| MKI67 | 1.269920335 | 0.836 | 0.076 | 0 |
| TYMS | 1.258634542 | 0.921 | 0.196 | 0 |
| GTSE1 | 1.258095181 | 0.838 | 0.065 | 0 |
| KNL1 | 1.211254305 | 0.808 | 0.07 | 0 |
| CCNB1 | 1.207464237 | 0.574 | 0.061 | 0 |
| ANP32E | 1.203158237 | 0.935 | 0.35 | 0 |
| TMPO | 1.189674304 | 0.963 | 0.372 | 0 |
| CENPE | 1.176546344 | 0.646 | 0.062 | 0 |
| ATAD2 | 1.175640309 | 0.879 | 0.186 | 0 |
| CKS2 | 1.162229207 | 0.915 | 0.352 | 0 |
| SMC2 | 1.15996713 | 0.946 | 0.293 | 0 |
| CENPU | 1.15920246 | 0.923 | 0.158 | 0 |
| EZH2 | 1.146514634 | 0.908 | 0.242 | 0 |
| BIRC5 | 1.145538214 | 0.884 | 0.108 | 0 |
| SGO2 | 1.142141597 | 0.736 | 0.089 | 0 |
| SMC4 | 1.139655197 | 0.878 | 0.226 | 0 |
| MAD2L1 | 1.134737829 | 0.912 | 0.174 | 0 |
| CLSPN | 1.114982192 | 0.783 | 0.126 | 0 |
| HIST1H1E | 1.091110429 | 0.783 | 0.242 | 0 |
| NUF2 | 1.089771137 | 0.805 | 0.081 | 0 |
| DLGAP5 | 1.07649419 | 0.659 | 0.037 | 0 |
| PBK | 1.067544717 | 0.871 | 0.096 | 0 |
| SPC25 | 1.055797351 | 0.813 | 0.078 | 0 |
| ESCO2 | 1.049837883 | 0.816 | 0.075 | 0 |
| CCNB2 | 1.046500487 | 0.634 | 0.062 | 0 |
| FBXO5 | 1.026336244 | 0.807 | 0.122 | 0 |
| SYNE2 | 0.99811688 | 0.894 | 0.3 | 0 |
| CKAP2L | 0.997605574 | 0.746 | 0.053 | 0 |
| CDKN3 | 0.994614502 | 0.739 | 0.085 | 0 |
| ECT2 | 0.992887122 | 0.742 | 0.089 | 0 |
| H2AFX | 0.98885675 | 0.911 | 0.323 | 0 |
| DHFR | 0.988260424 | 0.799 | 0.184 | 0 |
| SGO1 | 0.983176188 | 0.741 | 0.067 | 0 |
| HMMR | 0.978164402 | 0.565 | 0.045 | 0 |
| CENPH | 0.960884748 | 0.824 | 0.153 | 0 |
| HMGB3 | 0.957477872 | 0.908 | 0.321 | 0 |
| MFAP4 | 0.942515661 | 0.723 | 0.094 | 0 |
| CDK1 | 0.939867521 | 0.753 | 0.087 | 0 |
| C21orf58 | 0.926995381 | 0.757 | 0.093 | 0 |
| LMNB1 | 0.924452355 | 0.823 | 0.166 | 0 |
| DBF4 | 0.90682555 | 0.745 | 0.193 | 0 |
| CENPK | 0.90679132 | 0.81 | 0.137 | 0 |
| RAD51AP1 | 0.90493583 | 0.794 | 0.119 | 0 |
| TACC3 | 0.90060876 | 0.724 | 0.078 | 0 |
| PCNA | 0.893646656 | 0.813 | 0.253 | 0 |
| CRNDE | 0.888840089 | 0.816 | 0.181 | 0 |
| NDC80 | 0.885651313 | 0.703 | 0.057 | 0 |
| CKS1B | 0.884165644 | 0.796 | 0.219 | 0 |
| KIFC1 | 0.882481995 | 0.698 | 0.048 | 0 |
| ORC6 | 0.867309243 | 0.803 | 0.151 | 0 |
| FAM111A | 0.849146749 | 0.731 | 0.159 | 0 |
| DTYMK | 0.848179977 | 0.883 | 0.314 | 0 |
| CCNA2 | 0.847726876 | 0.668 | 0.052 | 0 |
| BARD1 | 0.84274872 | 0.784 | 0.196 | 0 |
| CDCA3 | 0.840762924 | 0.612 | 0.043 | 0 |
| NCAPG | 0.832625625 | 0.707 | 0.072 | 0 |
| PIMREG | 0.832314082 | 0.696 | 0.06 | 0 |
| KIF4A | 0.830302058 | 0.63 | 0.048 | 0 |
| KIF14 | 0.82972707 | 0.542 | 0.042 | 0 |
| HIST1H2AL | 0.819186382 | 0.602 | 0.046 | 0 |
| MND1 | 0.798285883 | 0.691 | 0.078 | 0 |
| HJURP | 0.796983593 | 0.629 | 0.032 | 0 |
| FOXM1 | 0.793549766 | 0.703 | 0.063 | 0 |
| FANCI | 0.772134423 | 0.705 | 0.084 | 0 |
| ATAD5 | 0.764954618 | 0.73 | 0.142 | 0 |
| GMNN | 0.762046965 | 0.772 | 0.199 | 0 |
| ASRGL1 | 0.759980531 | 0.823 | 0.25 | 0 |
| CCDC34 | 0.755442826 | 0.744 | 0.16 | 0 |
| CNTLN | 0.748645086 | 0.74 | 0.193 | 0 |
| HIST1H3G | 0.747455356 | 0.53 | 0.012 | 0 |
| HIST1H2AJ | 0.743987332 | 0.52 | 0.017 | 0 |
| KIF23 | 0.743848423 | 0.575 | 0.035 | 0 |
| KIF11 | 0.735388715 | 0.617 | 0.057 | 0 |
| KIF22 | 0.731576357 | 0.757 | 0.175 | 0 |
| KIF2C | 0.724414093 | 0.586 | 0.039 | 0 |
| HIST1H2AG | 0.720752444 | 0.574 | 0.049 | 0 |
| LINC01551 | 0.720261588 | 0.753 | 0.169 | 0 |
| RRM1 | 0.719794469 | 0.755 | 0.205 | 0 |
| HELLS | 0.71142263 | 0.676 | 0.146 | 0 |
| RACGAP1 | 0.710983159 | 0.645 | 0.084 | 0 |
| DIAPH3 | 0.704656766 | 0.614 | 0.067 | 0 |
| CHEK1 | 0.698246931 | 0.701 | 0.144 | 0 |
| AURKB | 0.695366908 | 0.596 | 0.042 | 0 |
| RFC3 | 0.692050125 | 0.692 | 0.128 | 0 |
| DTL | 0.690759123 | 0.585 | 0.072 | 0 |
| MELK | 0.69060853 | 0.654 | 0.066 | 0 |
| CENPM | 0.68857775 | 0.684 | 0.107 | 0 |
| BRCA2 | 0.687545082 | 0.621 | 0.099 | 0 |
| MYBL2 | 0.685813704 | 0.624 | 0.077 | 0 |
| KIF15 | 0.676065171 | 0.621 | 0.066 | 0 |
| HIST1H2BH | 0.672143187 | 0.58 | 0.043 | 0 |
| BUB1B | 0.672088196 | 0.562 | 0.042 | 0 |
| GGH | 0.667240173 | 0.774 | 0.214 | 0 |
| NCAPD3 | 0.660590239 | 0.694 | 0.166 | 0 |
| MPPED2 | 0.645411832 | 0.676 | 0.137 | 0 |
| NCAPG2 | 0.62556285 | 0.625 | 0.104 | 0 |
| BRCA1 | 0.618809493 | 0.624 | 0.124 | 0 |
| MXD3 | 0.6058879 | 0.556 | 0.056 | 0 |
| VRK1 | 0.585597683 | 0.643 | 0.122 | 0 |
| MIS18BP1 | 0.816196209 | 0.775 | 0.227 | 1.30E-298 |
| SEZ6 | 0.616178066 | 0.75 | 0.183 | 3.12E-297 |
| FRS2 | 0.903533003 | 0.856 | 0.288 | 3.47E-294 |
| GNG4 | 0.780924048 | 0.863 | 0.31 | 4.70E-290 |
| USP1 | 0.833742692 | 0.871 | 0.347 | 1.43E-289 |
| SAE1 | 0.604770444 | 0.702 | 0.19 | 2.24E-288 |
| HIRIP3 | 0.61519459 | 0.726 | 0.204 | 1.64E-287 |
| PEG10 | 0.806385673 | 0.907 | 0.338 | 3.87E-283 |
| NSD2 | 0.678577817 | 0.777 | 0.246 | 7.11E-281 |
| DRAXIN | 0.586822879 | 0.698 | 0.179 | 2.30E-274 |
| BMP7 | 0.70630407 | 0.801 | 0.236 | 3.61E-274 |
| AGAP2-AS1 | 0.851029909 | 0.878 | 0.308 | 1.60E-267 |
| PIK3R3 | 0.619062014 | 0.83 | 0.271 | 7.56E-258 |
| HDGFL3 | 0.648362589 | 0.882 | 0.333 | 6.03E-256 |
| B4GALNT1 | 0.87535187 | 0.918 | 0.388 | 1.13E-248 |
| MCM7 | 0.678388542 | 0.813 | 0.287 | 3.87E-247 |
| CDKN2A | 0.834328272 | 0.972 | 0.452 | 1.73E-242 |
| IGFBP2 | 0.623759076 | 0.99 | 0.459 | 4.23E-233 |
| CDKN2C | 0.7548806 | 0.866 | 0.358 | 2.83E-224 |
| EGFR | 0.694275214 | 0.96 | 0.442 | 2.69E-219 |
